# Molecular Engineering of *E. coli* Bacterioferritin: A Versatile Nanodimensional Protein Cage

**DOI:** 10.3390/molecules28124663

**Published:** 2023-06-09

**Authors:** Anton M. van der Ven, Hawa Gyamfi, Uthaiwan Suttisansanee, Muhammad S. Ahmad, Zhengding Su, Robert M. Taylor, Amanda Poole, Sorina Chiorean, Elisabeth Daub, Taylor Urquhart, John F. Honek

**Affiliations:** Department of Chemistry, University of Waterloo, Waterloo, ON N2L 3G1, Canada; anton.m.vanderven@gmail.com (A.M.v.d.V.); hawagyamfi@gmail.com (H.G.); uthaiwan.sut@mahidol.ac.th (U.S.); dr.sheeraz@uaar.edu.pk (M.S.A.); zhengdingsu@hbut.edu.cn (Z.S.); robert.taylor@easternhealth.ca (R.M.T.); amanda.n.poole0@gmail.com (A.P.); chiorean@ualberta.ca (S.C.); bhonek@rogers.com (E.D.); t.urquhart@outlook.com (T.U.)

**Keywords:** bacterioferritin, encapsulation, bioconjugation, heme, gold, green fluorescent protein, surface modification, host–guest, transglutaminase, in silico docking

## Abstract

Currently, intense interest is focused on the discovery and application of new multisubunit cage proteins and spherical virus capsids to the fields of bionanotechnology, drug delivery, and diagnostic imaging as their internal cavities can serve as hosts for fluorophores or bioactive molecular cargo. Bacterioferritin is unusual in the ferritin protein superfamily of iron-storage cage proteins in that it contains twelve heme cofactors and is homomeric. The goal of the present study is to expand the capabilities of ferritins by developing new approaches to molecular cargo encapsulation employing bacterioferritin. Two strategies were explored to control the encapsulation of a diverse range of molecular guests compared to random entrapment, a predominant strategy employed in this area. The first was the inclusion of histidine-tag peptide fusion sequences within the internal cavity of bacterioferritin. This approach allowed for the successful and controlled encapsulation of a fluorescent dye, a protein (fluorescently labeled streptavidin), or a 5 nm gold nanoparticle. The second strategy, termed the heme-dependent cassette strategy, involved the substitution of the native heme with heme analogs attached to (i) fluorescent dyes or (ii) nickel-nitrilotriacetate (NTA) groups (which allowed for controllable encapsulation of a histidine-tagged green fluorescent protein). An in silico docking approach identified several small molecules able to replace the heme and capable of controlling the quaternary structure of the protein. A transglutaminase-based chemoenzymatic approach to surface modification of this cage protein was also accomplished, allowing for future nanoparticle targeting. This research presents novel strategies to control a diverse set of molecular encapsulations and adds a further level of sophistication to internal protein cavity engineering.

## 1. Introduction

Substantial efforts are being made to utilize multimeric, self-assembling protein complexes in bionanotechnological applications [1,2,3,4,5]. Nanodimensional cage-proteins such as ferritins [6,7], cavity-containing enzymes [8], chaperonins [9], virus capsids [10,11,12], vault proteins [13,14], microbial microcompartments [15,16,17], and encapsulins [18] can be engineered with some precision to accommodate guest molecules within their hollow interior cavities. The strategic design of protein cages has resulted in the creation of biomolecular platforms capable of encapsulating a diverse set of guest molecules ranging from drugs [8,19], metal nanoparticles [20,21], enzymes [22,23], polymers [24,25], and oligonucleotides [26]. Applications of such systems to the areas of electrical device fabrication [27,28,29], tissue and cellular imaging [30,31], drug delivery [32,33], and metal nanoparticle generation [34] have been reported. Research on nanodimensional protein cages continues to generate more complex macro-scale arrangements, which will inevitably lead to additional applications [35,36,37]. Importantly, new encapsulation strategies capable of the *controlled and selective* introduction of guest molecules into the vacant cavities of these cages are a quintessential requirement for continuing advances in this area. 

**Figure 1:**
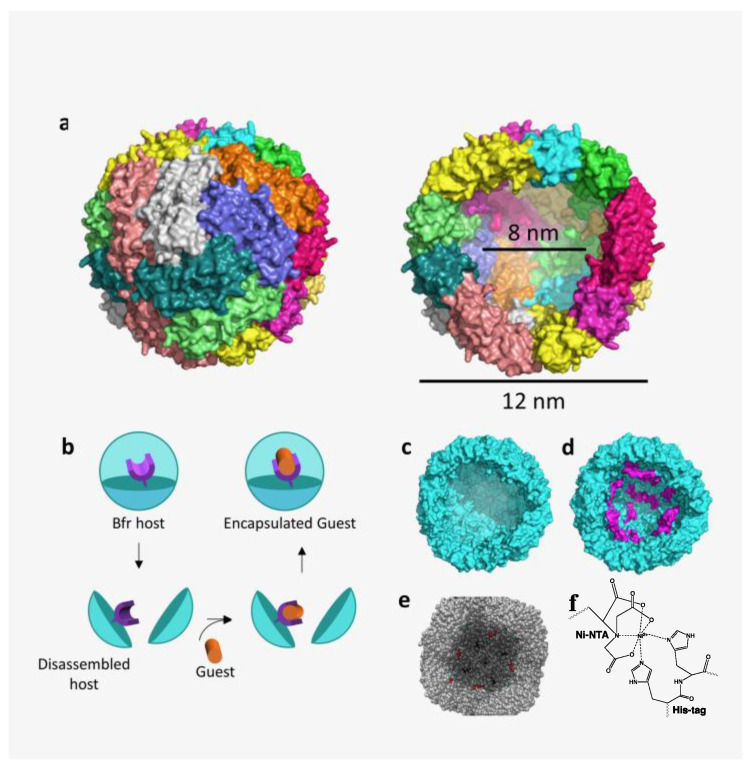
A structural and schematic overview of *E. coli* Bfr and the steps involved in the encapsulation of foreign guests within the engineered Bfr host scaffold. (**a**) Bacterioferritin (PDB: 1BFR generated with Pymol^TM^) highlighting the surface composition of subunits and the interior space with dimensions in nanometers. (**b**) The steps involved in encapsulation require a specific affinity pair, dissociation of the subunits, and exposure of the inner affinity partner to the guest molecule containing the complementary affinity partner, followed by subunit reclustering with entrapment of the target guest molecule. (**c**) Wild-type (WT) Bfr with the interior exposed. (**d**) Energy minimized interior of Bfr with the C-terminal His-tags represented in purple. (**e**) WT Bfr with the interior exposed showing some of the heme cofactors colored in red to clarify their positions in the cavity. (**f**) Chemical structure of the nickel (II) nitrilotriacetic acid (Ni-NTA)-His-tag interaction employed in this study.

*Escherichia coli* bacterioferritin (Bfr) is a member of the ferritin superfamily of iron storage proteins (Figure 1a) [38,39,40]. Ferritin family proteins have attracted the attention of researchers interested in the use of these protein cages for bionanotechnology purposes [41,42]. Commercially available non-engineered ferritins are frequently used for these purposes [43,44,45,46]. Bfr has clear distinguishing features from other ferritin family proteins and these differences provide Bfr with some important advantages. Bfr is composed of 24 identical subunits and contains up to twelve heme cofactors, each sandwiched between two neighboring protein subunits (Supplementary Materials: BacterioferritinMovie.mov) [47]. These two important structural differences from the ferritins, which lack heme and may be composed of two different subunits, lend Bfr to modification and assembly homogeneity, and to multiple ways of functionalizing the heme cofactors. Bfr subunits have C- and N-termini that are directed to the interior and exterior surfaces, respectively, of the Bfr protein capsule, thus providing two unique genetically modifiable surface positions. Dimensionally, Bfr is an approximately spherical shell with interior and exterior diameters of 8 and 12 nm, respectively (Figure 1a). Based on the potential for exploitation of these Bfr molecular characteristics, strategies were developed to fabricate a *highly controllable* nanodimensional Bfr scaffold. 

Initial strategies focused on engineering an affinity tag to the internal C-terminus of Bfr, a strategy that might be expected to control modified guest encapsulation (Figure 1b). Specifically, the Bfr interior was engineered by genetically fusing a hexa-histidine (His-tag) amino acid sequence at the C-terminus of the Bfr subunits to generate a Ni^2+^-dependent affinity interaction with guest molecules that contained a nitrilotriacetic acid (NTA), a well-understood affinity interaction (Figure 1f) [48,49]. Three structurally diverse guest molecules—a small fluorescent dye, a tetrameric protein, and a gold nanoparticle—were specifically encapsulated under controlled conditions. An encapsulation strategy was devised (Figure 1b), which required declustering of the engineered Bfr under non-denaturing conditions, affinity-selective complex formation between guest and declustered Bfr, and subsequent protein reclustering. Although the declustering and reclustering of capsule proteins such as ferritins has been found to be a successful approach to encapsulate a range of guest molecules, the affinity approach described herein was expected to lend itself to a more controllable entrapment strategy [4,7,50,51]. The first guest that was explored by this approach was a small molecular weight NTA-linked fluorescent dye, Pro-Q^®^ Sapphire 365 [52,53]. The second guest was a fluorescein-labeled globular tetrameric protein, streptavidin (SF), approximately 5 nm in diameter, which binds the cofactor biotin [54,55]. This was chosen to probe the capabilities of Bfr to accommodate complex guest molecules, such as proteins. In addition to a large biological molecule, a large inorganic 5 nm gold nanoparticle (AuNP) was also investigated to probe engineered Bfr host encapsulation capabilities. NTA surface-modified AuNP was chosen as a paradigm for the encapsulation of inorganic nanoparticles, such as plasmonic materials [56]. In addition to the study of a variety of encapsulated non-native guest molecules, the heme cofactor present in Bfr was modified with fluorescent dyes. This strategy was utilized to demonstrate the capability of this heme cassette to further functionalize and control the Bfr internal cavity as the heme propanoic acid side chains are pointed *toward* the inner cavity of Bfr (Figure 1e) (Supplementary Materials: HemeOrientation.mov). Furthermore, heme cofactors were attached to NTA moieties and employed for the encapsulation of an N-terminal His-tagged green fluorescent protein (GFP) [57]. Extended studies on surface modification of Bfr and in silico screening of potential ligands capable of replacing heme and altering the quaternary structure of Bfr were also investigated. Described below are the results of the engineering strategies to produce a *single* Bfr platform capable of the encapsulation of a wide range of guest molecules, as well as the generation of a heme-cassette tactic to further extend the capabilities of the Bfr nanoplatform.

## 2. Results and Discussion

Motivation for this work was driven by two factors: an ever-expanding need to produce novel materials with diverse functionalities from self-assembling biomolecules, and the need to further understand how to incorporate additional sophistication into biological architectures. The goal was to design several structural features into the Bfr scaffold to provide additional versatility to the ferritin class of protein cages, a focus of great current interest [7,58,59,60]. The implementation of engineered non-native affinity linkers in the interior of Bfr and the presence of chemically modifiable heme cofactors has demonstrated that the interior surface can be controllably modified to encapsulate a diverse set of molecular guests all the while employing the *same scaffold*.

### 2.1. Internal His-Tagged Bfr

The gene coding for *E. coli* Bfr [61] was cloned into a pET 22b vector to express both wild-type (WT) and His-tagged Bfr in *E. coli* (Supplementary Materials; Figures S1 and S2). The His-tag, which included a two amino acid linker (Leu-Glu) between the C-terminus and the His-tag, was fused onto the C-terminus, which is located on the internal surface of Bfr. His-tag Bfr was analyzed in silico to evaluate the extent of cavity space reduction that might occur by the presence of the His_-_tag (Figure 1c,d). The cavity was modestly reduced in diameter by approximately 1.5 nm with the placement of the new, but flexible, His-tag sequences. Possible size restrictions to the interior from extending His-tags for certain guest molecules led to the utilization of hybrid WT/His-tag Bfr combinations when necessary to alleviate and optimize any possible internal space restrictions when investigating guest encapsulations. Therefore, optimal ratios of WT to His-tag subunits were empirically evaluated for each guest molecule. In addition, a variety of conditions were employed to determine the optimal conditions for declustering and reclustering of the Bfr cage with minimal protein unfolding. In our studies, the presence of heme was a contributing factor to the formation/maintenance of the 24-mer quaternary Bfr structure (Supplementary Materials, Figure S3). In the absence of added heme, the C-terminal His-tag on individual subunits, which were overproduced by *E. coli* overexpression, could be strategically employed for affinity purification (see Section 3).

#### 2.1.1. Encapsulation of Dye Molecules within His-Tagged Bfr

**Figure 2:**
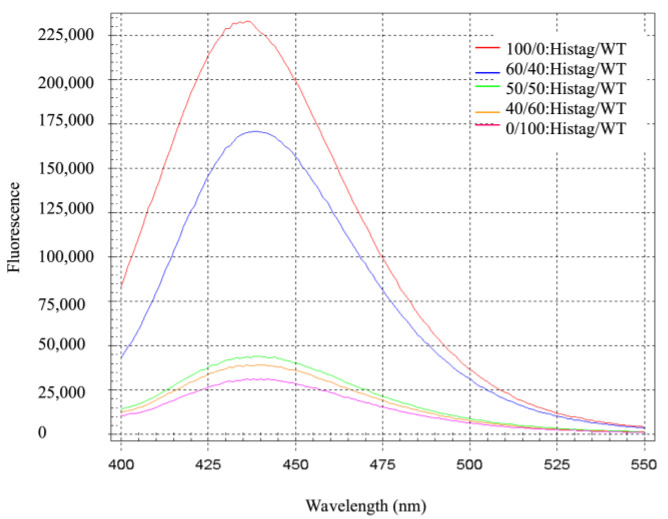
An increase in the amount of associated relative fluorescence units (RFU) was observed as a function of His-tag Bfr subunits in the isolated intact 24-mer complex, indicating the presence of the NTA-linked Pro-Q^®^ Sapphire 365 encapsulated dye. The presence of the affinity tag interaction was a critical component for the efficient encapsulation of the guest dye molecule to occur. No fluorescence was associated with the protein capsule in the absence of subunits lacking the His-tag fusion.

The capability of incorporating fluorescent dyes into bionanoparticles for eventual use in tissue and tumor imaging is an important application and has engendered great interest [62,63,64,65]. Hence, initial investigations were undertaken to explore the flexibility of various design principles to controllably accommodate small molecular weight fluorescent probes within the Bfr cavity. A commercial NTA-linked fluorescent dye (Pro-Q^®^ Sapphire 365) [52,53], used in His-tag protein staining, was utilized as an initial test for controlled encapsulation within an engineered His-tagged Bfr using the His_6_-NTA affinity interaction. To expose the inner surface of Bfr to guest molecules such as the Pro-Q^®^ Sapphire dye, declustering of Bfr was performed using a change of pH to 2.0, which was determined to thoroughly disrupt quaternary structure, but not tertiary structure as monitored by dynamic light scattering (DLS). After incubation with the dye in the presence of Ni^2+^ and followed by dialysis to pH 7.0 to allow for reclustering, the correct diameter of the resulting 24-mer Bfr with associated fluorescence was confirmed utilizing dynamic light scattering (DLS) and size exclusion chromatography (Supplementary Materials, Figure S4). The presence of the dye was confirmed by analysis of the resulting fluorescence properties of the dye–Bfr complex and the results demonstrated that dye encapsulation was dependent on the presence of the His-tag (Figure 2). Evidence for some fluorescence quenching of the encapsulated dye was also observed (Supplementary Materials, Figure S5). By establishing the importance of affinity interactions with this model dye, the validation of this system to readily encapsulate larger and more complex guest molecules was made, which was followed by a more detailed study. 

#### 2.1.2. Encapsulation of Proteins within His-Tagged Bfr

**Figure 3:**
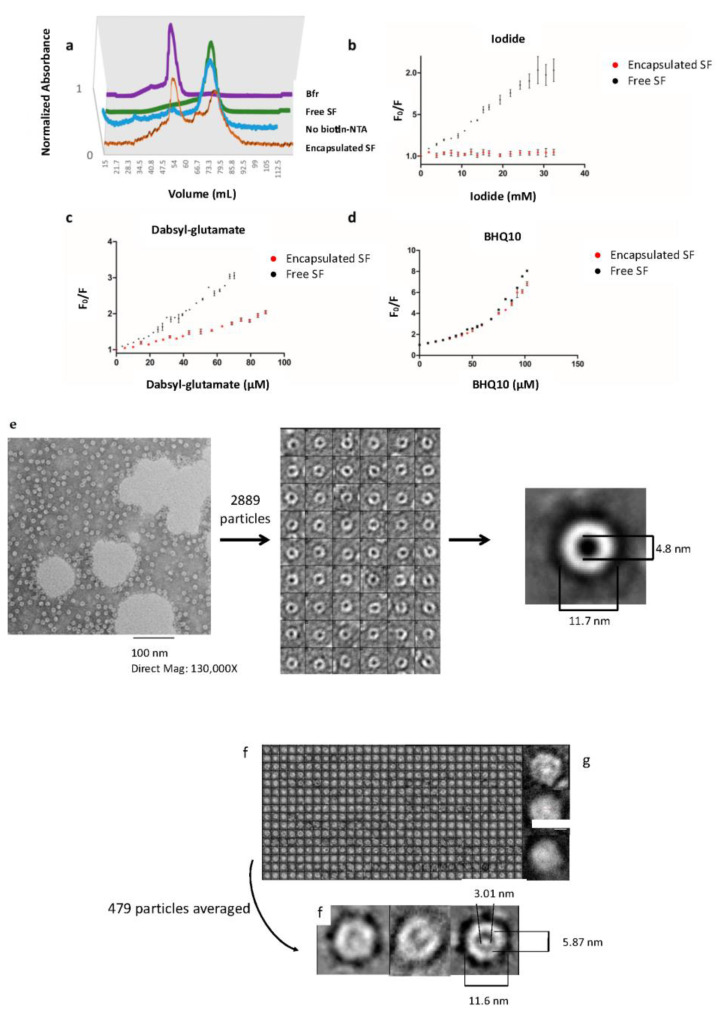
Encapsulated fluorescein-labeled SF in His-tag: WT (60:40) Bfr was analyzed using multiple fluorescence and microscopic methodologies. (**a**) Encapsulated SF was purified using size-exclusion chromatography (GE Sephacryl^TM^ S-300HR resin) to separate encapsulated SF from free SF. Bfr was detected using the specific 418 nm marker for the heme cofactor. SF was detected using the fluorescein excitation wavelength of 495 nm. The encapsulated SF was observed to have an identical elution time to Bfr in the presence of the biotin NTA linker. Without the biotin NTA linker (negative control), SF eluted at the same time as free SF. Fluorescence quenching experiments (**b**–**d**) were performed with iodide, dabsyl-glutamate, and BHQ^®^10, respectively [67,68,69,70,71]. Free SF was observably quenched to a greater extent than the encapsulated SF, and thus consistent with encapsulation protecting the SF guest. Empty Bfr and Bfr that had encapsulated SF were both analyzed utilizing TEM (**e**–**g**). (**e**) Empty Bfr was analyzed using TEM with molybdic acid, and it was observed that the negative stain could penetrate the interior space of Bfr and aid in the visualization of the internal cavity. Single particle analysis of empty Bfr exhibited a spherical particle that was approximately 12 nm in diameter, which was the appropriate size for Bfr. (**f**) TEM images of encapsulated SF were distinguished by the observable feature in the interior of Bfr, which was the presence of an excluded zone of negative stain, as seen with multiple single particles. Single particle analysis of the encapsulated SF enhanced the observed stain-excluded interior delineated by a thin coating of stain (insert in (**f**)). (**g**) Highly resolved and unprocessed images showed stain-excluded zones in SF encapsulated within Bfr as well.

To explore the capabilities of engineered Bfr to accommodate complex protein cargo, a fluorescein-labeled streptavidin (SF) was utilized to track and analyze encapsulation within Bfr. Streptavidin has one of the highest known non-covalent association constants with its native ligand, biotin [54]. To provide a strong affinity link between streptavidin and the internal surface of His-tagged Bfr, NTA-linked biotin was utilized. Tetrameric streptavidin is exceptionally stable to many denaturants, and thus guanidinium hydrochloride was evaluated for its ability to act as a possible alternate Bfr declustering reagent which could prove useful for some future applications of the Bfr platform [55]. The optimal ratio for His-tag:WT was empirically determined to be 60:40. Encapsulated SF was purified employing size exclusion chromatography monitoring the excitation maxima for the fluorescein on SF and the absorbance maxima for heme to specifically track the elution of each protein. When tracking the elution of encapsulated SF, an earlier elution time was observed compared to non-encapsulated SF (Figure 3a). These results are entirely consistent with what has been observed when monitoring the encapsulation of green fluorescent protein within the cavity of lumazine synthase using size-exclusion chromatography [66]. Control experiments lacking a biotin NTA intermediary linker demonstrated an elution profile typical of free SF. This indicated that SF was indeed associating with Bfr and that both the His-tag and biotin NTA were essential for host–guest association to occur. Further characterization was undertaken to elucidate the nature of this interaction.

Confirmation of the internalization of the SF into Bfr was evaluated using fluorescence quenching studies (Figure 3b–d). Three quenching agents were used to evaluate encapsulation: iodide, dabsyl-glutamate and Black Hole Quencher^®^ 10 (BHQ^®^10) [67,68,69,70,71]. Experimental and theoretical data were used to calculate a theoretical Forster resonance energy transfer (FRET) distance for both dabsyl-glutamate and BHQ^®^10 with SF (Supplementary Materials) [72]. FRET distances were approximately 4 and 5 nm for dabsyl-glutamate and BHQ^®^10, respectively. Analysis of the X-ray structure of Bfr [47] provided an estimation of the protein shell width to be between 2.5 and 3 nm without solvation radius, electrostatics, or other factors considered.

Both encapsulated and non-encapsulated SF was titrated with a quencher and again separately with buffer to control for dilution effects and plotted as Stern–Volmer plots (Figure 3b–d) [73]. The encapsulated SF exhibited little to no fluorescence quenching over the course of titration with the collisional quenching agent, iodide, whereas dabsyl-glutamate exhibited modest levels of quenching, and BHQ^®^10 showed essentially complete quenching, the latter two both being FRET-based quenching agents. These results are entirely consistent with the expectation of the relative quenching capabilities of dabsyl-glutamate and BHQ^®^10 when compared to the theoretical FRET distances and the width of the Bfr protein shell.

Visual confirmation of SF encapsulation was made by employing transmission electron microscopy (TEM). This technique has been successfully utilized to visualize encapsulated guest molecules within protein cages [74,75]. Two TEM-negative stains were used, uranyl acetate and molybdic acid, to highlight Bfr and determine the presence of internalized guest molecules within the cage [76]. Due to the intrinsic pores (5.5 Å in diameter) found in the structure of Bfr, the atom-sized stains would be able to penetrate the core, as evidenced by virus-like particles [77,78]. Optimization of the penetration of the negative stains across the native pores of Bfr was determined by TEM analyses (Figure 3e). The highest resolved, unprocessed images showed a guest particle in the interior of Bfr with a diameter of approximately 5.4 nm (Figure 3g). This matched the expected diameter of the tetrameric structure of SF, consistent with SF encapsulation. Processing of a total of 479 particles from multiple encapsulated SF images, using the software package EMAN2 ver. 2.07 [79], generated an average image of encapsulated SF in Bfr (Figure 3f). In this case, the processed images had an outer diameter consistent with that of native Bfr of 11.6 nm, while the inner cavity contained a particle of approximately 3.7 nm in diameter, although processing artifacts may have occurred to alter the calculated guest diameter (Supplementary Materials Figure S6). In summary, the results of the size exclusion, fluorescence quenching, and TEM experiments are entirely consistent with SF encapsulation within the engineered Bfr scaffold. 

#### 2.1.3. Encapsulation of a Gold Nanoparticle (AuNP) within His-Tagged Bfr

**Figure 4:**
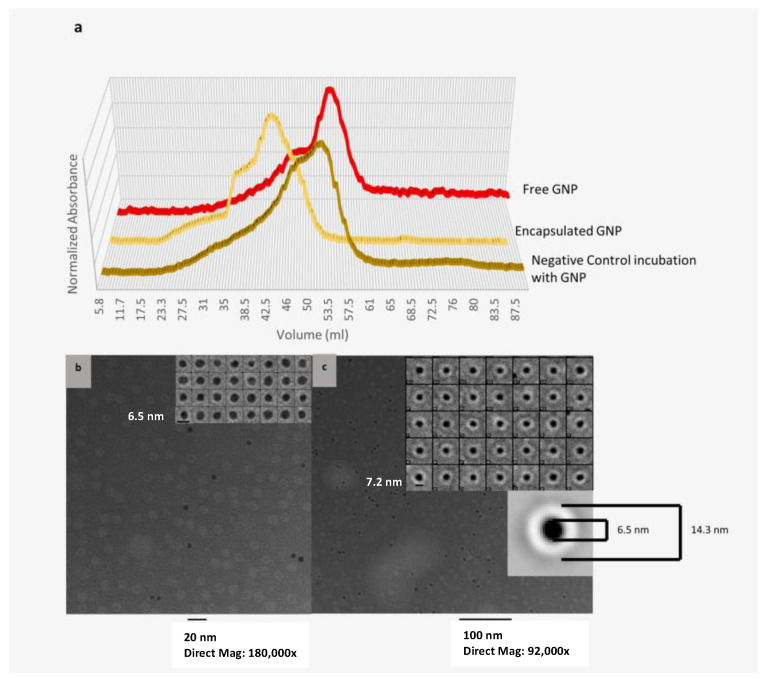
Purification and characterization of encapsulated AuNP in 100% His-tag Bfr. (**a**) Separation of encapsulated AuNP from free AuNP was performed via size exclusion chromatography (GE Sephacryl ™ S-300HR resin). AuNP was monitored by its absorption maximum of 518 nm, and Bfr was monitored by the heme absorption maximum of 418 nm. The encapsulated AuNP clearly eluted earlier than free AuNP, and the presence of the His-tag was crucial for this association as the use of 100% WT Bfr, which lacked the His-tag, failed to encapsulate the AuNP (negative control incubation with AuNP). (**b**,**c**) The WT control and His-tag encapsulated Bfr were examined using TEM. (**b**) TEM analysis of the WT Bfr/AuNP control clearly showed that the AuNP was not encapsulated and that the polymer coating on the AuNP was not as thick as the protein coat as seen in (**c**). (**c**) TEM analysis of the His-tag Bfr encapsulation of AuNP clearly exhibited a thick protein coat covering all AuNP in the images.

To evaluate the ability of the Bfr capsule to accommodate metal clusters, a pre-formed gold nanoparticle was studied as a potential guest. For AuNP encapsulation, 5 nm NTA polymer-coated AuNPs were employed. An alternate declustering strategy, that of temperature, was utilized. A temperature increase to 70 °C was found to be sufficient to decluster the protein cage. The optimal ratio of His-tag:WT for this encapsulation was empirically determined to be 1:0. Confirmation of encapsulated AuNP within Bfr was explored using size exclusion chromatography and TEM. Encapsulation of AuNP in the presence of His-tag Bfr, as monitored by the 518 nm maximum absorbance wavelength of AuNP, resulted in a shift in the elution time for AuNP suggesting a host–guest interaction (Figure 4a). When the same encapsulation conditions were performed in the absence of His-tag Bfr (100% WT Bfr) as a control, there was no observed shift in the elution profile of AuNP.

TEM was performed on both encapsulated and free AuNP. All the AuNPs present in these images were encapsulated within Bfr. Single particle analysis was performed as before using 4364 single particles (Figure 4). Slight increases in the external diameter of the host–guest complex were observed with an increase of approximately 2–3 nm based on the averaged image. Such increases suggested slight conformational changes to the protein coat structure to accommodate this extremely large guest molecule, estimated to contain approximately 3000 gold atoms (Supplementary Materials) [80,81,82,83]. These stable conformational changes were allowable as a likely result of the compensatory number of multiple affinity interactions between host and guest.

### 2.2. Heme-Dependent Cassette Strategy

Modification of the native heme cofactor was explored as a means to further functionalize the inner surface of Bfr in a cassette-like strategy. The heme associated with Bfr is sandwiched between two neighboring subunits, a total of twelve heme cofactors are present in a fully heme-saturated Bfr 24-mer. The Fe^2+^ in the heme is bound to the methionine sulfur atom (Met52) from each of the two neighboring subunits [84,85,86]. Of interest to this work is the orientation of the two propanoic acid side chains of the heme groups, which are directed into the inner cavity of Bfr (Figure 1e; Supplementary Materials: HemeOrientation.mov) [47]. Therefore, it was hypothesized that control of the inner cavity and even guest cargo incorporation might be further modulated by replacing the natural heme cofactor with heme analogs carrying the desired guest molecule(s).

#### 2.2.1. Heme Modification and Reinsertion into Bfr

**Figure 5:**
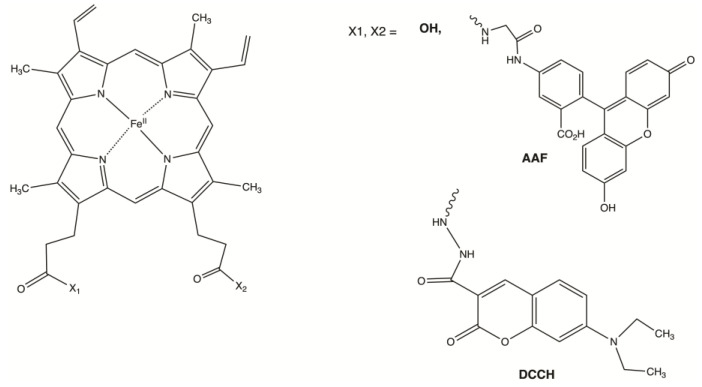
Dye–heme analogs employed in this investigation. Mono and bis AAF-heme analogs were synthesized from activated NHS-heme using 5-(aminoacetamido)fluorescein (AAF). Mono- and bis- DCCH-heme analogs were synthesized from activated NHS-heme and 7-(diethylamino)coumarin-3-carbohydrazide (DCCH).

The heme-dependent cassette strategy was first examined by the specific functionalization of heme with fluorophores. Two fluorophores were examined as the intended labels. The coumarin derivative 7-(diethylamino)coumarin-3-carbohydrazide (DCCH), and the fluorescein derivative 5-(aminoacetamido)fluorescein (AAF) were used to chemically label the heme propanoic acid side chains after activation of the carboxylic acids with resin immobilized carbodiimide (PS-carbodiimide) and N-hydroxysuccinimide (NHS) (Figure 5 and Supplementary Materials Figure S7). Reactions of these fluorophores with the heme were monitored by analysis of the resulting products by mass spectrometry (Supplementary Materials; Figure S8). After reaction completion, the fluorophore-modified hemes were purified and exchanged with the native heme present in Bfr [86]. The reconstituted Bfr samples were then purified by size exclusion chromatography and analyzed by electrospray mass spectrometry to confirm the presence of the dye–heme analogs (Supplementary Materials; Figures S9 and S10). 

Modified hemes reconstituted in place of native heme in Bfr exhibited red-shifted Soret bands, indicative of the bis-methionine coordination of heme within Bfr (Supplementary Materials Figure S11a–c) [86]. The percent incorporation of AAF-heme was approximately 100%. This was determined using the heme extinction coefficient and comparing the concentration to that of Bfr, which was determined using the Bradford protein assay. Due to low solubility in water, the DCCH-heme was incorporated into Bfr at very low, but still detectable, amounts. Bfr complexes incorporating either DCCH or AAF-modified heme were studied utilizing native polyacrylamide gel electrophoresis to demonstrate the maintenance of proper protein quaternary structure in the presence of fluorescently labeled heme (Supplementary Materials Figure S11d). Fluorescent bands were visible at the exact positions on the gel that were stained by the protein-specific Coomassie stain.

In addition to the spectral and native polyacrylamide gel electrophoresis (PAGE) experiments, quenching and fluorescence anisotropy experiments were also performed to confirm the proper incorporation of modified heme within Bfr (Supplementary Materials Figure S12). The results of these studies indicated larger fluorescence anisotropies for protein-incorporated modified hemes (Supplementary Materials). It was observed that the AAF-heme incorporated within Bfr exhibited the same fluorescence quenching pattern as the encapsulated SF. The Stern–Volmer plot confirmed that the fluorophore was protected to the same extent as the encapsulated SF. Fluorescence anisotropy plots (Perrin plots) were also consistent with incorporation [73]. Taken together, the above experiments are consistent with the ability of Bfr to accommodate chemically-modified heme analogs. 

#### 2.2.2. Bis-NTA-Heme Enables Encapsulation of His-Tagged Proteins into Bfr

The ability to replace the heme cofactor in Bfr with analogs was investigated as to its ability to reverse the “polarity” of the encapsulation process such that an NTA-labeled Bfr could be used to encapsulate His-tag protein guests. One strategy to accomplish this was the possibility that modified heme, carrying the NTA functionality, might be capable of providing the affinity interaction between the His-tag protein guest and the Bfr subunits when declustered. Hence, heme was coupled to N^α^,N^α^-bis(carboxymethyl)-l-lysine using the above heme modification methods to provide the desired bis-NTA-heme analog. The bis-NTA heme was obtained in approximately 70% yield (Supplementary Materials, Figures S13 and S14). Purified bis-NTA-heme was reconstituted into Bfr using the previous methods. The incorporation of bis-NTA-heme into Bfr itself was monitored using the spectral shift from 380 to 418 nm as was seen previously with heme and modified heme-fluorescein incorporation into Bfr. 

**Figure 6:**
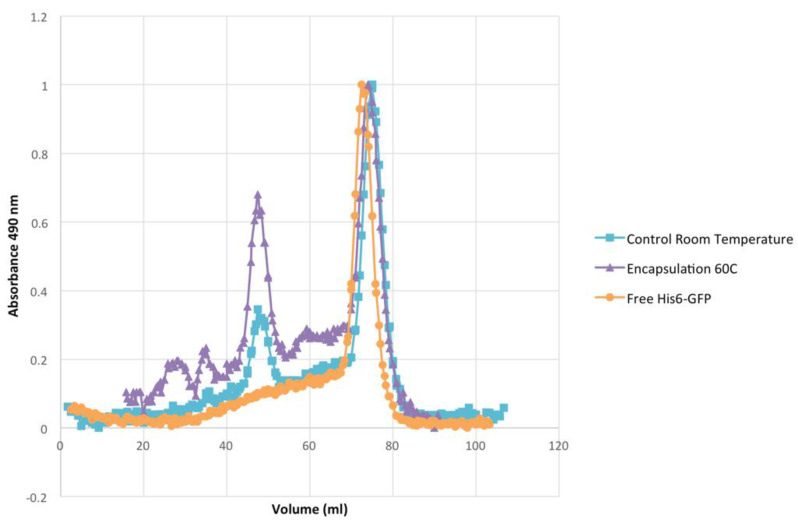
Chromatogram of three separate experiments run on a Sephacryl S-300 HR gel permeation resin. The wavelength monitored during elution of protein was 490 nm, the excitation wavelength for the purified His-tag GFP. Free His-tag GFP was run as a control, with a single elution peak corresponding to the protein at ~75 mL. Second, a run of encapsulated His-tag GFP within WT Bfr with the incorporated bis-NTA-heme. This was incubated at 60 °C for 10 min and cooled for 30 min to room temperature before injection on to resin. Finally, a control of the above condition without heat treatment was plotted.

**Figure 7:**
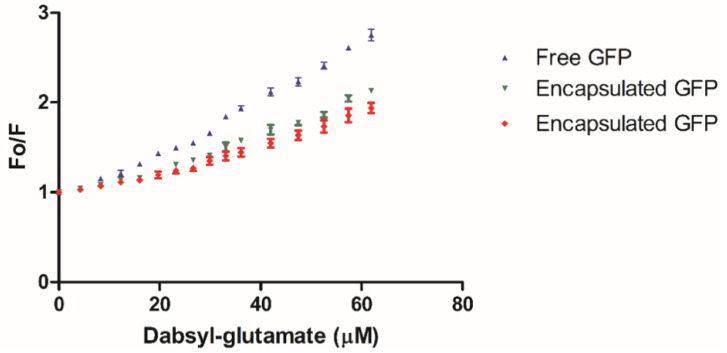
Quenching experiments with dabsyl-glutamate performed with encapsulated His-tag GFP (two trials shown) and free His-tag GFP. The control (free His-tag GFP) exhibited enhanced quenching compared to the encapsulated His-tag GFP with dabsyl-glutamate.

**Figure 8:**
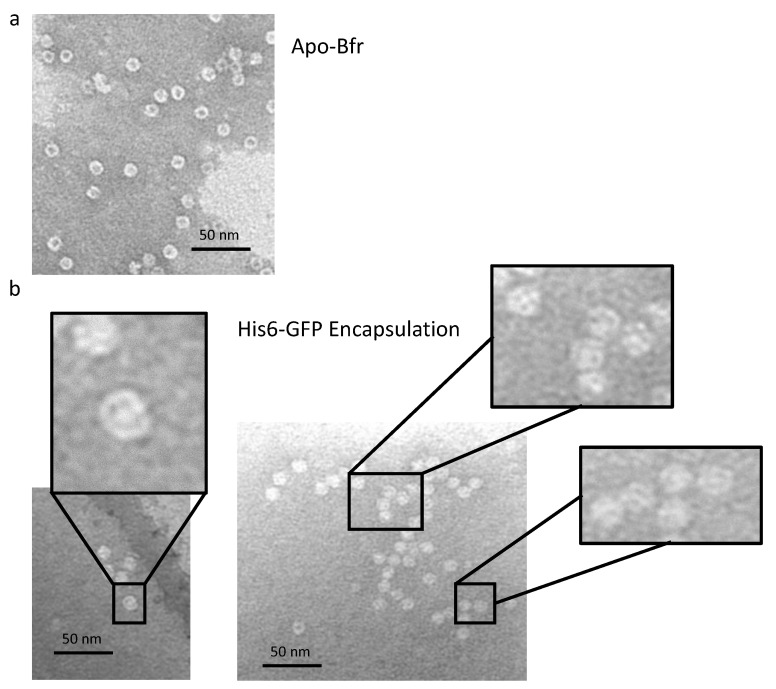
Encapsulation of His-tag GFP within WT Bfr was imaged using TEM. (**a**) Wild-type Bfr (without His-tag GFP added) was imaged with stain (darker core) penetration allowing for visualization of the empty core; (**b**) TEM images of encapsulated His-tag GFP within bis-NTA-Bfr with stain being excluded from the occupied core of Bfr due to the presence of the guest His-tag GFP.

N-Terminal His-tagged green fluorescent protein (His-tag GFP) was employed as a model His-tagged guest protein to study guest encapsulation using the bis-NTA-heme cassette (Supplementary Materials; Figure S15). The thermostability of purified His-tag GFP [87,88] was measured in terms of its ability to retain fluorescence with exposure to higher temperatures for 10 min in low and high sodium chloride solutions. The temperatures used were 60, 70, and 80 °C. It was observed that the fluorescence of His-tag GFP at 60 °C for 10 min did not result in a loss of fluorescence, whereas both 70 and 80 °C heat treatments for 10 min resulted in minor and total loss of fluorescence, respectively, in both buffer conditions. His-tag GFP encapsulation was accomplished utilizing the temperature method as described for AuNP encapsulation. Encapsulation was observed to occur after several trials by first incorporating bis-heme-NTA into WT Bfr, followed by the addition of Ni^2+^ ion, with the subsequent addition of the His-tag GFP and mixing at 60 °C for 10 min. Analyses of (i) the resulting gel permeation chromatograms against those of the starting components (Figure 6); (ii) the fluorescence quenching studies (Figure 7); and (iii) the TEM visualization experiments (Figure 8) are consistent with the GFP guest being encapsulated within the Bfr cavity. Native gel electrophoresis was also consistent with GFP fluorescence being observed in the Bfr gel band (Figure S16). His-tag GFP was not encapsulated when WT Bfr with unmodified heme was employed, indicating the essential nature of the His-tag-NTA interaction for encapsulation.

### 2.3. Chemoenzymatic Surface Modification Strategies for Future Targeting Capabilities of Bfr

With the successful development of strategies to encapsulate a diverse range of molecular guests into the hollow cavity of Bfr, attention turned towards the development of bioconjugation reactions on the Bfr outer surface. Successful approaches here could be important in providing future targeting capabilities of cargo-laden Bfr nanoparticles to cellular targets as well as enabling non-peptide modifications to be explored. One strategy that has been employed successfully to direct nanoparticles to target cells has been the chemical bioconjugation of an antibody or small molecule receptor ligands such as riboflavin or folate to the nanoparticle’s surface [89,90,91,92]. The benefit of a method that employed a selective chemoenzymatic approach for surface modification would be that the *same* protein cage could be *differentially* modified with various targeting ligands and, hence, be targeted to different cells depending on the need. In the case of Bfr and chemical bioconjugation, the drawback is that multiple surface-exposed lysine residues (Lys 2, 6, 13, 76, 99, and 143) are present on each subunit of Bfr (a total of 144 exposed lysines on each capsule), providing a challenge in controlling the number and position of the address surface labels. As previously mentioned, Bfr and other members of the ferritin family have subunit termini oriented such that the N-terminus of each subunit is exposed on the outer surface of the capsule and the C-terminus is exposed on the inner surface (see earlier sections). Standard recombinant DNA methods have been successfully employed to provide fusion peptide sequences on the outer surface of human and horse ferritins and in one case, Bfr itself which has an affinity for tumor cells [58]. In this specific strategy, that of employing a specific surface peptide fusion, a decision as to the target for the nanoparticle would have to be made early in the design process, and each target would need to have a different DNA sequence employed and, hence, completely different capsule proteins would need to be prepared each time. This strategy could certainly be utilized and has been successful in targeting a cargo Bfr with the disulfide-cyclized peptide, RGD4C, a peptide with affinity for various tumor cells [58]. An alternate strategy that could utilize the *same* Bfr construct but allow for *different surface modifications* through a chemoenzymatic approach might provide yet another tactic with increased versatility. In the current investigation, this approach has been found to be successful in the surface modification of Bfr and details on transglutaminase surface modification of Bfr are provided below.

#### 2.3.1. Outer Surface Peptide Sequence Design 

This strategy involved the introduction of a peptide fusion at the N-terminus located on the outer surface of Bfr with peptide sequences recognized by the enzyme transglutaminase (TGase). This approach would permit the site-specific attachment of various amine-containing molecules to a displayed surface peptide containing a glutamine residue as the enzyme can replace the nitrogen of the side chain amide in glutamine with a diverse set of amines [93,94,95]. The result is the overall conversion of the glutamine to a glutamine analog. The enzyme has been shown to accept a wide range of amines as co-substrates and can result in the attachment of fluorophores, peptides, antibodies, and azide/acetylene-containing amines useful in advanced click chemistry bioconjugation strategies. The sequence surrounding the target glutamine is important in substrate recognition by the enzyme, although a definitive sequence pattern has yet to be found [96,97]. Horse spleen ferritin has been shown not to be a substrate [98] for bacterial transglutaminase, although it has several glutamines exposed on its surface. In the case of Bfr (PDB: 1BFR), Gln 72, 142, and 147 are exposed on the surface of the capsule. Our attempts to modify glutamine residues on either horse spleen ferritin or E. coli Bfr using transglutaminase and the fluorescent amine, dansylcadaverine, likely failed due to a combination of the lack of appropriate surface exposure of the target glutamines, lack of the required conformational flexibility of the protein regions associated with these glutamines, and/or poor substrate recognition sequences. It was clear based on these preliminary studies that peptide fusions were required to better present a target glutamine to the enzyme, as well as to ensure that an appropriate glutamine-containing peptide sequence would be available to the enzyme. Based on previous studies which have attempted to define transglutaminase substrate recognition sequence(s), a series of N-terminal peptide fusions, or “Qtags” on C-terminal His-tagged Bfr were investigated as potential substrates for the enzyme (Table 1). Further included is a peptide fusion capable of serving as a Sortase A substrate sequence, which could employ the enzyme sortase A instead of transglutaminase for chemoenzymatic bioconjugation [99]. Qtag1 and Qtag2 also contained a modified S-peptide sequence, which is known to act as a substrate for transglutaminase and could potentially be employed in reconstituting RNAse A activity on the surface of Bfr when incubated with the RNAse S protein [100]. However, this additional property will not be discussed further in this report. It is interesting to note that the reverse approach has been reported; that is, the lysine residues on a recombinant human H ferritin can be attached using TGase to an anti-EGFR nanobody displaying a Qtag sequence [101].

#### 2.3.2. Site of Transglutaminase Fluorophore Bioconjugation

The ability of Qtags1–4 to act as substrates towards transglutaminase were investigated by incubation of each recombinant protein with 5-dimethylaminonaphthalene-1-(*N*-(5-aminopentyl))sulfonamide (dansylcadaverine), in the presence of the *Streptomyces mobaraensis* transglutaminase to generate fluorescently labeled Bfr. Labeling conditions were optimized for the Qtag1 construct employing 15% SDS-PAGE gel electrophoresis and visualized for fluorescence gels (Supplementary Materials, Figure S17). Analysis of product fluorescence spectra and electrospray mass spectrometry of the purified proteins confirmed that Qtag1-Bfr and the other Qtag constructs are TGase substrates (Supplementary Materials, Figures S18 and S19). His-tag Bfr constructs with the additional Qtags as well as the sortase tag were all labeled with dansylcadaverine in the presence of TGase. Bacterioferritin constructs without the Qtag were not labeled by the TGase. The absence of dansyl cadaverine labeled bands when just His-tagged Bfr was used validates the necessity of the Qtag constructs for chemoenzymatic bioconjugation of this protein cage. 

To confirm the position of TGase labeling, the Qtag1-containing Bfr was reacted with cyanogen bromide (CNBr) to prepare peptide fragments suitable for peptide sequencing by mass spectrometry. The number of fragments that can be generated from Qtag1-Bfr was estimated with the PeptideCutter tool to obtain the fragmentation pattern, which resulted in nine peptide fragments with even distribution of all the glutamines in the Bfr subunits and the tags (Supplementary Materials, Figure S20). The results of these studies were consistent with Gln12 in Qtag1 being the covalent attachment site for dansylcadaverine. Expected sites for modification for the other Qtags based on the previous literature studies of favorable Gln sites are shown in Table 1. 

#### 2.3.3. Quaternary Structure Dependence on Surface Peptide Sequence 

**Figure 9:**
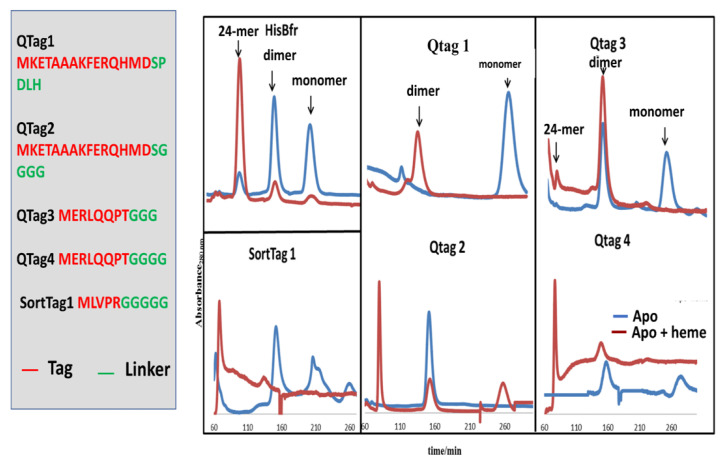
Effect of the presence or absence of N-terminal Qtag or SortTag peptide fusions on Histag-Bfr quaternary structure as measured by size-exclusion chromatography (Sephacryl^TM^ S-300 HR) in the presence or absence of heme. Size-exclusion chromatography was used to separate the various quaternary structures (monomer, dimer, 24-mer). The blue curves represent protein solutions without heme and the red curves are the heme-reconstituted samples.

To make these surface peptide fusions useful in protein cage engineering, it was critical to confirm that these peptide fusions would not affect the 24-mer quaternary structure of His-tagged Bfr. The effects of peptide fusion on His-tagged Bfr quaternary structure formation were investigated by employing size-exclusion chromatography with these constructs in the absence and presence of heme. Figure 9 presents these findings.

It was clear from analysis of the size-exclusion chromatography experiments that linker sequence and length were critical factors in allowing the proper quaternary structure of His-tagged Bfr to be attained in the presence of the N-terminal fusion tags. Four or more glycine residues in the linker region, such as Qtag2, Qtag4, and SortTag1 were able to self-assemble into the proper 24-mer quaternary structure in the presence of heme (Figure 9). The more sterically demanding linker present in Qtag1 or the shorter (Gly)_3_ linker present in Qtag3 failed to allow for proper self-assembly. In the structure of bacterioferritin (PDB:1BFR), Met1 is adjacent to two bulky residues, Lys2 and Glu66, which likely contribute to the necessity of a non-sterically demanding linker to obtain the proper quaternary structure (24-mer) of the protein in the presence of heme. Attempts to open up the surface area surrounding the N-terminal methionine by making the Qtag2 His-tagged Bfr muteins E86A (corresponding to E66 in wild-type Bfr lacking Qtag2), K22A (corresponding to K2 in wild-type Bfr lacking Qtag2), and the double mutant E86A/K22A resulted in the absence of intact 24-mers being formed even after heme addition. Hence, modifications of either of these two residues were not found to be beneficial to the accommodation of more sterically demanding surface tags.

#### 2.3.4. Transglutaminase Bioconjugation on an Intact HostG–Uest Supramolecular Complex

The host–guest AuNP (5 nm)-protein complex (AuNP-His-tag Bfr-Qtag2) (Supplementary Materials; Figure S21a) was dansylated employing the transglutaminase strategy to illustrate that Bfr can be modified on the outer surface while a guest is encapsulated within the protein cage. AuNP encapsulated His-tag Bfr-Qtag2 was prepared and incubated with dansylcadaverine in the presence of microbial TGase. The incubation mixture was then run on a Sephacryl^TM^ S-300 HR size-exclusion column to separate the dansylated protein cage from other oligomers in the solution (Figure S21b). The desired fractions were analyzed on a native-PAGE gel to observe a dansylated 24-mer protein band, while TEM was employed to visualize the encapsulated AuNP. SDS-PAGE electrophoresis was employed to detect fluorescently labeled Bfr subunits (Figure S21c,d). These experiments confirm the proof-of-concept that engineered Bfr can be chemoenzymatically surface labeled and in the future could be repurposed for targeted cargo delivery where the cargo could be a drug or a magnetic resonance imaging (MRI) agent, and the exterior could be modified with cell recognition peptides, antibodies or aptamers.

### 2.4. In Silico Screening for Heme Cofactor Replacements

Lastly, it was of interest to determine if small molecules other than heme could be utilized by Bfr to control its quaternary structure. Initially, several heme analogs were to be explored in reconstitution experiments: ferriprotoporphyrin IX (heme, the natural Bfr cofactor); protoporphyrin IX (natural cofactor lacking the Fe^2+^ metal); protoporphyrin IX Zn(II); and protoporphyrin IX dimethyl ester. It had previously been shown [58] that protoporphyrin IX Zn(II) is able to serve in establishing the proper Bfr quaternary structure, and this analog was to be employed, along with ferriprotoporphyrin IX, as positive controls in reconstitution experiments. In addition, in silico screening was employed to identify possible non-heme-based molecules capable of controlling Bfr quaternary structure, an area not previously investigated. Additionally, molecular scaffolds capable of binding to the heme sites in Bfr and providing the necessary quaternary structure to the protein could be employed in the future to design cytotoxic agents capable of being transported within the heme binding site and providing therapeutic use when released into the target cells.

#### 2.4.1. In Silico Screening

Two high-throughput in silico docking strategies were employed to screen an Aldrich Market Direct^®^ Phase Database (version Q2 2018; containing 7.2 million molecules with >19 million variants which included conformers, charge states, and tautomers for each molecule). The ligand-based approach entailed the mapping of key functional group interactions between the already bound heme cofactor and neighboring protein interactions in the heme binding site in Bfr (PDB:1BFR). The fragment-based approach employed the removal of the heme cofactor from its Bfr binding site, followed by the generation of a grid encompassing the heme binding site protein residues. This binding site was then screened against a small library of small molecules termed fragments (667 small molecules default and range from methanol to thiophene and a complete listing is available at Schrodinger, LLC., New York, NY, USA) to map out the internal cavity of the heme binding site with functional groups without any bias to heme-containing molecules. Subsets of these fragments were combined to generate the “fragment-based” pharmacophore. The generated ligand-based and fragment-based pharmacophores (Figures S22 and S23) were then each separately screened against the curated seven million compound Phase database using Phase software (Schrodinger, LLC.) [102,103].

**Figure 10:**
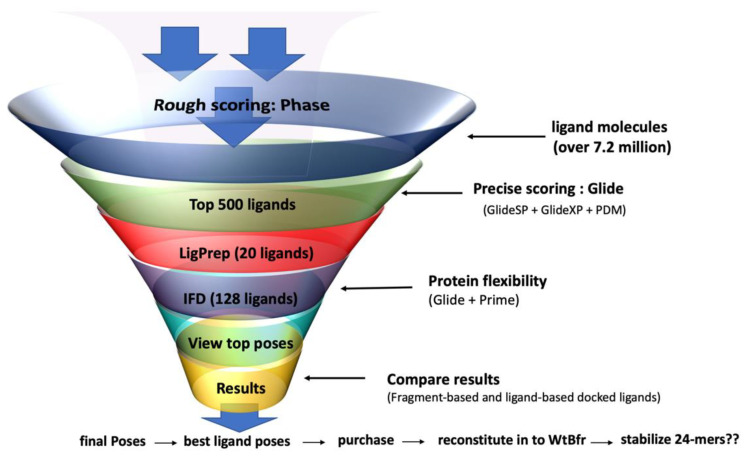
Docking funnel summarizing the workflow for screening the molecular database against the ligand-based and fragment-based heme binding site pharmacophores.

An overview of the screening workflow employed for each pharmacophore type is presented in Figure 10. Initially, a rough scoring function in Phase Docking software (Release version 2019-4; Schrodinger, LLC.) was employed to reduce the molecule database to the top 500 ligands capable of pharmacophore docking and this was done for each pharmacophore. Molecules were sorted by the number of interactive sites, volume score, and their overall effective interactions. The database employed was the Aldrich Market Direct^®^ Phase Database (version Q2 2018), which contains 7.2 million ligand molecules (up to 19.1 million conformers/tautomers/charge states) with molecular weights between 250 to 500 Da. Each of the pharmacophores contained seven interactive sites, and a condition of the Phase screening required that a molecule must match at least four of the seven interactive sites of the pharmacophore before it can be classified as a hit.

The top 500 hits were retained for each screen (fragment-based and ligand-based). It was interesting to observe that the 5 top-scoring hits obtained from the Phase screen for both pharmacophores were structurally related to protoporphyrin IX, the natural heme cofactor for Bfr. The top 500 Phase-screened ligands were then selected for Standard Precision Glide (Glide SP) docking, followed by an Extra Precision Glide scoring protocol [104,105,106]. Glide SP and XP dockings were performed on the hits to obtain a set of molecules that optimized their fit into the Bfr receptor site. A receptor grid was employed for this second stage of screening. Met52, the Fe^2+^-ligand to the heme, was observed to contribute unfavorably to the interaction energies in most cases. Phe26 was quite favorable in both ligand- and fragment-based results despite its size and it was involved in either pi–pi or pi–cation interactions with most of the top ligands. This observation is consistent with the work reported by Nam and coworkers who observed that Trp26 at the heme binding site of bacterioferritin from *Rhodobacter sphaeroides* is an essential residue for stabilizing the heme molecule in that bacterioferritin [107]. The most favorable residue at the binding site of Bfr was determined to be Asn23, which was involved in hydrogen bonding (donor/acceptor) with the high-ranking docked molecules.

**Figure 11:**
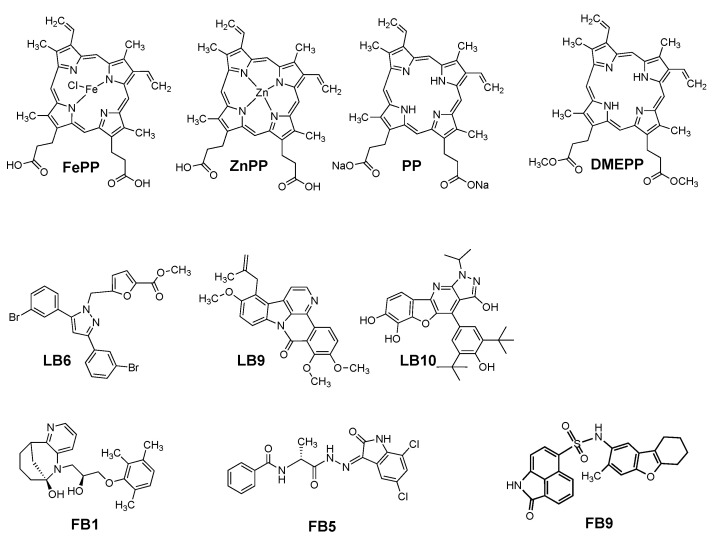
Chemical structures of the ligands studied for their ability to replace heme in the control of the Bfr quaternary structure.

The above screening stage is a rapid set of protocols utilizing rigid docking protocols but with soft van der Waals contacts. The two sets of ligands were separately docked into the same Bfr rigid receptor to obtain their Glide scores. To account for the Bfr receptor flexibility, the Induced Fit Docking (IFD) (Schrodinger, LLC) strategy was employed [108,109]. The Induced Fit Docking protocol, which allows for both protein residue and small ligand rotation/translation changes in the docking protocol, was run selecting the top 15 tight binders, 3 moderate binders, and 2 weak binders from the Glide XP ranking. The 20 ligands were prepared to employ LigPrep (Schrodinger, LLC) to generate each ligand’s corresponding set of low-energy 3D structures; overall, 128 structures were obtained and used for IFD using the IFD protocol in Maestro (Schrodinger, LLC). The above was undertaken for each of the two pharmacophore models. The IFD score was then used to rank the ligands into tight binders, moderate binders, and weak binders. Samples of several of the higher-ranked compounds were purchased and studied further with Bfr with respect to their ability to replace heme in being able to generate the 24-mer quaternary structure. Overall, 13 ligands were investigated: four protoporphyrin analogs (Protoporphyrin IX Fe (II) (heme; FePP); Protoporphyrin IX Zn (II) (ZnPP); Protoporphyrin IX molecule (PP); and Protoporphyrin IX dimethyl ester (DMEPP)), and six from the two in silico pharmacophore screenings: LB6, LB9, LB10 (ligand-based), and FB1, FB5, and FB9 (fragment-based), and three amino acids (Tyr, Asp, Glu) were also screen as ligands that would be predicted not to have any influence on the protein’s quaternary structure (Figure 11).

#### 2.4.2. Effect of Various Non-Heme Ligands on Bfr Quaternary Structure

The ability of the various ligands to act as reasonable replacements of the heme cofactor in Bfr and aid in the formation of the 24-mer capsule was investigated using His-tag Bfr as this construct is an optimized Bfr useful in future applications. Reconstitution experiments were set up initially with the four protoporphyrin analogs by combining 1 molar equivalent of WTBfr with 2 molar equivalents of the ligands at 80 °C for 10 min each and allowing the mixtures to cool down to room temperature. The mixtures were then buffer-exchanged into 150 mM NaCl, 50 mM MOPS or HEPES-pH 7.5 and filtered through a 0.45 μm nylon syringe filter. Dynamic light scattering (DLS) analysis was used to analyze the sample for quaternary structures in the solution after reconstitution. Analysis of this qualitative screen indicated that FePP and ZnPP were the most capable of the heme analogs to shift the equilibrium of the protein towards a 24-mer quaternary structure in this type of screen (Figure S24). This is consistent with previous research on the ability of ZnPP which has been shown to stabilize the Bfr quaternary structure. The DLS results for the ligand-based and fragment-based molecules, as well as the three simple amino acids, are shown in Figures S25–S27, respectively. Although preliminary, these initial results indicate the potential for small molecule manipulation of the quaternary structure of Bfr.

## 3. Materials and Methods

### 3.1. DNA Cloning and Manipulation

Recombinant WT and His-tag Bfr were generated and purified using standard molecular genetics and chromatographic techniques (Supplementary Materials). 

### 3.2. Computational Modeling

Computational modeling was performed using Sybyl software (Sybyl-X, version 3.1; Tripos Inc., St Louis, MO, USA) with the PDB file (1BFR) for *E. coli* bacterioferritin (Supplementary Materials). 

### 3.3. Encapsulation of Pro-Q^®^ Sapphire, SF, and AuNP

The optimum ratios of His-tag Bfr subunits to wild-type (non-His-tag) Bfr subunits were determined empirically for each guest molecule. 

His-tag Bfr exclusively, or various ratios of His-tag Bfr to wild-type (WT) Bfr at 0.5 mg mL^−1^ were dissolved in 10 mM piperazine-N,N′-bis(2-ethanesulfonic acid) (PIPES; pH 8.0), 150 mM NaCl, and 10% glycerol and the solution was acidified to pH 2.0 with acid and incubated at 23 °C for 90 min to decluster the protein, yet allow for subsequent reclustering without unfolding. The declustered protein was incubated subsequently with a commercial dye solution (50% *v*/*v*), Pro-Q^®^ Sapphire 365 oligohistidine gel stain for 3 h at 23 °C. The dye solution contained an NTA-linked coumarin fluorophore pre-complexed with Ni^2+^ ion. The solution was then dialyzed into 4 L of 10 mM PIPES, and 100 mM NaCl buffer at pH 8.0 for 24 h, followed by size-exclusion chromatography. The Pro-Q^®^ Sapphire 365 NTA dye has a maximum excitation wavelength of 345 nm and a maximum emission wavelength of 440 nm. 

A molar equivalent of a fluorescein-labeled streptavidin, termed SF, (0.1 mg, 1.89 × 10^−9^ mol) was pre-incubated with an NTA functionalized biotin, biotin-X-NTA (Supplementary Materials) (0.021 mg, 2.93 × 10^−8^ mol, 4.0 equiv.) and nickel sulfate (4.5 µg, 2.93 × 10^−8^ mol, 4.0 equiv.) in 50 mM Tris, 100 mM NaCl buffer at pH 8.0 for 10 min at 23 °C. This solution was then added to a mixture of 60% His-tag Bfr and 40% WT Bfr (0.84 mg, 1.89 × 10^−9^ mol, 1.0 equiv. total) in 8 M guanidinium hydrochloride buffered with 50 mM tris(hydroxymethyl)aminomethane (Tris), 100 mM NaCl at pH 8.0 and incubated for 90 min at 5 °C. The solution was then dialyzed against 4 L of 50 mM Tris 100 mM NaCl buffer at pH 8.0 for 24 h. 

Gold nanoparticles (AuNP; 5 nm), coated with a polymer displaying NTA functionalities (1.0 × 10^−10^ mol), were pre-incubated with nickel sulfate (2.0 × 10^−8^ mol, 200 equiv.) in 50 mM Tris, 100 mM NaCl buffer at pH 8.0 for 10 min at 23 °C. This solution was then mixed with 100% His-tag Bfr (0.1 mg, 2.0 × 10^−10^ mol, 2 equiv.) at 70 °C for 90 min. After 90 min, the solution was left to cool to 23 °C. 

Subsequently, size-exclusion chromatography was carried out for each of the above encapsulation systems (Supplementary Materials).

### 3.4. Fluorescence Analysis

Fluorescence quenching experiments were performed on a PTI fluorimeter A-1010B. Encapsulated Pro-Q^®^ Sapphire 365 oligohistidine gel stain was analyzed using slit widths set to 1 mm in the sample chamber. A small volume cuvette (Hellma 45 μL) was utilized with a path length of 0.3 cm. Quenching agents utilized were iodide, dabsyl-glutamate, and BHQ^®^-10, with concentrations of 0.1 M, 0.27 mM, and 0.39 mM, respectively. Dabsyl-glutamate was prepared from dabsyl-Cl following a previously reported procedure [69]. Each of these quenchers was titrated with one or two microliter volume increments. Anisotropy was measured on a Molecular Devices Spectramax M5 using the pre-set basic anisotropy protocol. Sucrose from a 60% (*w*/*v*) stock was added in 3% increments to a final concentration of 30% sucrose (Supplementary Materials).

### 3.5. TEM Preparation and Imaging

Two negative stains were used to generate the contrast needed to visualize the protein, molybdic acid ((NH_4_)_6_Mo_7_O_24_), and uranyl acetate (UO_2_(CH_3_COO)_2_). Molybdic acid and uranyl acetate stains were prepared as 1.0% and 0.5% solutions, respectively [74,110]. The preparation of grids and staining methods are elaborated further in the Supplementary Information (Supplementary Materials).

### 3.6. Heme Analogs

Bis-N-hydroxysuccinimide-heme (Bis-NHS-heme) was synthesized by the addition of hemin (1) (200 mg., 0.307 mmol) and PS-carbodiimide resin (954 mg, 1.3 mmol/g, 4.0 equiv.) to anhydrous dimethylformamide (3.2 mL) with stirring at 23 °C for 10 min (Supplementary Figure S7). N-Hydroxysuccinimide (NHS) (88 mg, 0.76 mmol, 2.5 equiv.) was added to this solution and stirred at 100 °C for an hour in a Biotage^®^ Initiator 8 microwave synthesizer (Biotage^®^ Inc., Charlotte, NC, USA). This resulted in the production of a mixture of mono (2) and bis (3) NHS-modified hemes. The mixture was used as such in the subsequent reactions.

#### 3.6.1. Reactions of Heme-NHS with Fluorescein, and Coumarin Derivatives 

7-(Diethylamino)coumarin-3-carbohydrazide (DCCH)-heme was synthesized by mixing the above NHS–heme mixture (5.5 mg, 6.79 µmol) and 500 µL of a 10 mg/mL solution of DCCH (4.67 mg, 16.97 µmol, 2.5 equiv.) into dimethylformamide (1.5 mL) stirred at 50 °C for 6 h in a Biotage^®^ Initiator 8 microwave synthesizer (Supplementary Materials, Figure S7). This resulted in the production of mono- (4) and bis- (5) DCCH-heme products. The reaction of 5-(aminoacetamido)fluorescein (AAF) with the NHS-activated heme mixture was similar to the DCCH reaction, with a total of 2.5 mg of AAF (6.17 µmol, 2.5 equiv.) being used. This resulted in the production of mono- (6) and bis- (7) AAF-heme products. Compounds were purified as described in Supplementary Materials.

#### 3.6.2. Insertion of Modified Heme into Bfr 

Mono-substituted DCCH and AAF modified heme (2.6 × 10^−8^ mol) were separately added to His-tag Bfr (1.3 × 10^−8^ mol of subunit dimer) at 80 °C in buffer (0.2 M 2-(N-morpholino)ethanesulfonic acid (MES), 1.0 M NaCl) at pH 6.5 for 10 min, employing a previously reported method for reconstitution of heme into Bfr [86]. After 10 min of incubation the sample was left to cool to 23 °C. Uncomplexed mono-substituted modified heme was separated from modified heme reconstituted Bfr utilizing gel filtration (Sephadex^®^ G-25; 50 mM Tris, 100 mM NaCl buffer; pH 8.0).

#### 3.6.3. Bis-NTA-Heme Synthesis 

The reaction scheme was derived using a Biotage^®^ PS-carbodiimide to activate the carboxylic acid groups on the heme and label them with an N-hydroxysuccinimide (NHS). Commercially available hemin (200 mg; 0.307 mmol) was dissolved in 3.2 mL of anhydrous dimethylformamide (DMF). Next, Biotage^®^ polystyrene-linked carbodiimide (PS-carbodiimide) (710 mg; 0.923 mmol) was added to the hemin and the solution was stirred for 5 min at RT. This was followed by the addition of N-hydroxysuccinimide (NHS) (123 mg; 1.07 mmol). The reaction mixture was placed in a microwave reactor and stirred for 1 h at 100 °C. Once complete, the mixture was filtered through glass wool and precipitated using 10× the volume of chilled isopropanol (−20 °C). The pellet was collected by centrifugation at 2500× *g* and dried *in vacuo*. The reaction success was checked using thin-layer chromatography (TLC) and mass spectrometry (MS). Once dry, the NHS-heme product was stored at −20 °C away from light until used.

Dried NHS-heme (10.3 mg; 0.127 mmol) was dissolved in 343 μL of DMF. Nα,Nα-Bis(carboxymethyl)-l-lysine (Lys-TA) (10 mg; 0.381 mmol) was dissolved in 1 mL of anhydrous dimethyl sulfoxide (DMSO) and this was added to the heme-NHS solution. The mixture was stirred in a microwave reactor for 1 h at 70 °C. The products of this reaction were again verified using TLC and MS (Supplementary Materials).

#### 3.6.4. Bis-NTA-Heme Purification 

To purify mono from bis-NTA-heme, the product was dried in vacuo to remove the DMF, and then re-suspended in a solution of 0.1 M NaOH with 50% mixture of methanol, and added to a 3 g C-18 samplet cartridge (Biotage^®^, Inc.), and left to dry. The samplet was run on a C-18 column with a gradient from 10 mM phosphate buffer pH 8.0 increasing to 100% methanol over a volume of 400 mL. Fractions were collected in 5 mL volumes, and the first eluting peak of around 150 mL was collected and dried in vacuo. The sample (1–10 mg) was re-suspended in distilled water (500 µL) and run on a P2 size exclusion column. The column was run in distilled water and the first eluted peak was collected and dried using a SpeedVac™ and stored at −20 °C away from light. The bis-NTA-heme dried sample was refrigerated and re-suspended for incorporation. 

### 3.7. His-Tag GFP Isolation and Purification 

The His-tag GFP was expressed in an *E. coli* BL-21 cell line from a pET plasmid using IPTG (kind gift from Professor Jeanne Hardy, Department of Chemistry, University of Massachusetts, Amherst). The plasmid was transformed into BL21 cell lines and then grown at 37 °C in 5 mL of LB media overnight. This starter culture was added to 1.5 L of LB media and grown to an O.D600 between 0.6–1.0 absorbance units. Then, IPTG was added to a final concentration of 1 mM and grown to shake at 200 rpm with a temperature reduced to 20 °C for 8–12 h. The cells were harvested using centrifugation at 10,000 rpm for 20 min. The pellet was re-suspended in 20 mL of buffered 50 mM Tris, 100 mM NaCl solution at pH 8.0, and then lysed in a homogenizer. The homogenized solution was centrifuged and the supernatant was collected. This solution was loaded onto an immobilized metal affinity chromatography column with 50 mM Tris, 20 mM imidazole, and 100 mM NaCl at pH 8.0. The target protein was eluted using the same buffer with 300 mM imidazole using a gradient from 0–100% elution buffer. The eluting fractions were evaluated with SDS-PAGE and the fractions containing pure target protein were collected and concentrated using a stirring Amicon concentrator with a 10,000 MW cut-off. This was flash-frozen and stored at −80 °C until required for use. The MW was confirmed using ESI mass spectrometry (Supplementary Materials).

### 3.8. Incorporation of Bis-NTA-Heme within Bfr

The bis-NTA-heme was suspended in a 0.2 M MES buffer. Bis-NTA-heme was mixed with WT Bfr in a ratio of 2:1. This was heated to a temperature of 80 °C for 10 min and allowed to cool to room temperature. To remove excess unbound bis-NTA-heme, the solution was concentrated using a spin column with a 10 kDa molecular weight cut off and washed 5 times with buffer.

### 3.9. Native PAGE

Native PAGE gels were prepared as stacks of 10% and 5% acrylamide gels. The lower third of the gel was 10%, while the upper two-thirds were 5% acrylamide gel. These gels were cast in Bio-Rad Mini-PROTEAN^®^ plates and run at 180 V for 20–30 min. Gels were analyzed under a gel doc with a UV plate. Protein bands in gels were stained using Coomassie Brilliant Blue R-250 

### 3.10. His-GFP Encapsulation 

His-tag GFP was added to WT Bfr solution in a 10:1 molecular ratio. The mixture was incubated for 10 min at 60 °C followed by a 30 min cooling period. The success of encapsulation was monitored by size-exclusion chromatography (SEC), fluorescence quenching, and transmission electron microscopy (TEM). The resultant mixture was separated on a Sephacryl™ S-300 size-exclusion column. Fractions for the first eluting peak were pooled, concentrated using a spin column with a 10 kDa MW cut-off, and prepared for further testing.

Fluorescence quenching was performed using Black Hole Quencher 10^®^ (BHQ10) and dabsyl-glutamate, at a concentration of 0.4 mM in 50 mM Tris, 100 mM NaCl, pH 8.0. An aliquot of 50 μL of sample was added to a 50 µL cuvette and the initial fluorescence was recorded. Next, quencher was added in 1 μL increments up to 10 µL followed by 2 µL additions until another 10 µL was added. Fluorescence was recorded after each addition. Samples were imaged using TEM and 0.5% molybdic acid negative stain, as described for SF encapsulation.

### 3.11. Transglutaminase Surface Modifications on His-Tag Bfr and Qtag-Surface Modified His-Tag Bfr

Qtags1–4 and Sortase1 surface-labeled His-tag Bfr constructs and microbial transglutaminase incubations on the overproduced proteins are detailed in Supplementary Materials. 

### 3.12. Dynamic Light Scattering (DLS) Experiments with His-Tag Bfr and Heme Replacements

DLS was used to monitor small-molecule mediated 24-mer formation in BFr. The samples were buffer exchanged into Bfr storage buffer (150 mM NaCl, l50 mM MOPS-pH 7.5). A 50 μL filtered sample was then placed into a 45 μL Hellma cuvette (with 0.3 mm path length) and run in triplicates on a Zetasizer Nano ZS model ZEN3500. The scattered light was collected at 90° with fiber optics and then converted to an electrical signal in an avalanche photodiode array (APDs), visualized in the form of intensity over time. Water was used as a dispersant with a refractive index of 1.33, and the temperature was set to 20 °C. Sizes were estimated from the Stokes–Einstein relationship (D = kBT/6πηRH), assuming that they all existed as spheres in solution and the dispersant viscosity (η) was set as the sample viscosity.

## 4. Conclusions

The ability to design and exquisitely control host–guest interactions for nano-sized biomolecular frameworks is critical to further advances in bionanomaterials. One area of focus in biomaterials research is the discovery and manipulation of nanodimensional proteins containing an empty cavity or core which can be “filled” with guest molecules [1,4,5,111]. These proteins are diverse and can perform not only their “normal” biological function but can be employed as “protein cages” and can provide internal cavities for encapsulation and materials science applications, as they can act as molecular containers for metal cluster synthesis or in drug delivery or as components in nanodimensional fabrication [20,42,51]. These proteins can be spherical virus-like nanoparticles such as the protein shell (capsid) of the MS2 bacteriophage or multisubunit cage-like proteins such as the iron storage protein ferritin, or the enzyme lumazine synthase, for example [6,7,42,112,113]. Research on new protein cages and strategies to control their cavities are critical aspects of innovation in this area. 

Previous studies have generally relied upon simple diffusion or electrostatic attraction to encapsulate guest molecules within these protein cages, directly or by declustering/reclustering the cage [51,114,115,116]. To create a more versatile and controllable cage, the present work develops new strategies to employ more complex ferritins, with the *Escherichia coli* bacterioferritin (Bfr) as a molecular scaffold. In addition to its 24 identical subunits, each of which has an N-terminus that is exposed on the *outer* surface of the cage and a C-terminus that is exposed on the *inside* surface, Bfr also contains 12 exchangeable heme groups sandwiched between adjacent subunits with their propanoic acid side chains pointed *into* the cavity [47,59,117]. (Supplementary Materials: BacterioferritinOverview.mov; HemeOrientation.mov). Narrow portals for metal ion ingress/egress are the only access routes to the inside. However, ferritins, including Bfr, can be declustered/reclustered under certain conditions of pH, temperature or additive [60]. 

Two new strategies were explored to control the encapsulation of a diverse range of molecular guests compared to random entrapment. The first was the design and incorporation of a histidine-tag peptide sequence fused to the C-terminus of the Bfr subunit (His-tag Bfr), as this would be displayed to the aqueous environment when the recombinant Bfr protein was declustered and available for an affinity interaction with a guest molecule that contained a nitrilotriacetate (NTA) functional group in the presence of Ni^2+^. This strategy proved successful despite the conditions required for declustering and reclustering the protein. NTA-containing fluorophores (Pro-Q^®^ Sapphire 365) were controllably entrapped within the cavity of His-tagged Bfr, whereas no evidence for dye encapsulation was found for Bfr lacking the His-tag Bfr. This approach could potentially provide a new approach to the synthesis of nanodimensional fluorescence imaging agents for medical applications once a targeting moiety was attached to the surface of the dye-encapsulated Bfr (dye@Bfr) (see below) [65,118,119]. A larger guest molecule, that of streptavidin, was then investigated. The His-tag Bfr construct was employed to controllably entrap a fluorescently labeled tetrameric protein, streptavidin (~52 kDa), in the presence of biotinamidocaproyl–nitrilotriacetic acid (Biotin-X NTA) as streptavidin has a high affinity for biotin. Various biophysical techniques were used to confirm encapsulation of size exclusion purified streptavidin@Bfr including fluorescence quenching of the fluorophore attached to the streptavidin, and transmission electron microscopy (TEM). To our knowledge, no reports have appeared that employ a His-tag-NTA interaction to encapsulate proteins within a cage protein and extend our knowledge of new strategies to entice complex biomolecules to serve as guest molecules. This approach differs from and is complementary to the recent report of ferredoxin (~8 kDa) encapsulation into bacterioferritin as the current method does not rely on random entrapment during the reclustering of the dissociated subunits but is controlled by the designed His-tag–NTA affinity interaction [7]. It is interesting to note that studies have found a modified avidin protein to be a component of a tumor chemotherapeutic wherein the avidin is injected into a tumor followed by a biotinylated radioactive compound, thus localizing the radioactive therapeutic within the tumor [120,121]. It might be suggested that a similar approach could be possible but involve the delivery of the (strept)avidin into a tumor by a targeted Bfr, with the release of the cargo into the tumor by endocytosis, and followed by injection of the radioactive biotinylated agent into the tumor. Further exploitation of the His-tag–NTA interaction for encapsulation was used to entrap a 5 nm gold nanoparticle coated with NTA functional groups. Encapsulated gold nanoparticles have been reported to be useful in bioimaging and medicine and new methods to fabricate these nanoparticles are of importance [20,21,122].

The second strategy was to make active use of the twelve heme cofactors situated between adjacent subunits in Bfr. They are uniquely poised with their propanoic acid moieties pointed towards the Bfr cavity (Supplementary Materials; HemeOrientation.mov). It was unknown as to what attachments could be made to the propanoic acid groups on the heme without weakening the effect of heme on the quaternary structure of Bfr. Hence, two fluorescent dye molecules (AAF and DCCH) were studied as attachments to explore this aspect of Bfr, as well as, if successful, provide another approach to fabricating fluorescently-labeled nanoparticles. The heme analogs were successfully synthesized and incubated with declustered Bfr followed by protein reclustering. Size-exclusion chromatography purified reconstituted Bfr particles were shown to indeed entrap the heme analogs (confirmed by mass spectrometry), exhibit fluorescence, and were further investigated by fluorescence quenching and fluorescence anisotropy experiments. The heme modification was extended to the attachment of N^α^,N^α^-bis(carboxymethyl)-l-lysine to the heme providing an NTA group, allowing for the reversal of the encapsulation strategy; that is how the His-tag would be situated on the guest molecule rather than the Bfr. This would be very useful in allowing for a wide range of proteins to be encapsulated as the His-tag peptide is a commonly employed protein affinity tag for recombinant proteins. Successful synthesis of the NTA-containing heme analog provided a new cofactor capable of use in encapsulating the His-tagged construct of the green fluorescent protein (GFP). Successful encapsulation of GFP was confirmed by analyzing the size exclusion purified GFP@Bfr by spectroscopic methods as well as TEM. This approach supplies a novel strategy to encapsulate His-tagged proteins of less than 8 nm in overall dimension (approximate size of the internal cavity of Bfr).

To endow targeting capabilities to the Bfr nanoparticles, a strategy was investigated that would be flexible and capable of attachment of a range of targeting peptides/antibodies/ligands to the Bfr outer surface. This would, therefore, require that the attachment of the targeting moiety be done *subsequent* to the recombinant production of the Bfr protein to avoid having to recombinantly produce many different targeting Bfr molecules for a range of applications. Transglutaminase chemoenzymatic attachment of amine-containing ligands to the side chain of glutamine residues was chosen as one approach to generating such a flexible approach. Experiments were undertaken to uncover an optimized linker to the outer N-terminus of the Bfr subunit as well as contain a transglutaminase recognition sequence having the target glutamine available for reaction. It was found that a linker containing four or more glycine residues was critical to maintaining the proper 24-mer quaternary structure of Bfr. Successful attachment of a model amine, dansylcadaverine, was attained and confirmed by detection of the fluorescence of the labeled subunits after polyacrylamide gel electrophoresis on the purified protein. It is proposed that a range of amine-containing ligands and proteins could be attached to the surface of Bfr by this approach and serve as future targeting agents for medical applications. Limitations of this approach would be the need for the presence of a suitably sterically unencumbered amino group on the ligand, which may need to be engineered into the ligand.

Lastly, it was of interest to explore the possibility that select small molecules might be able to replace the heme cofactor and also favor the proper quaternary structure of Bfr. This would be of interest not only to ameliorate our fundamental knowledge of heme binding sites in general but also might identify the heme binding sites of Bfr as novel drug binding sites, allowing for certain types of therapeutic agents to be carried within Bfr employing these sites. An in silico docking screen of the Aldrich Market^®^ Direct Phase Database was undertaken using the heme binding sites as the docking site to be screened for potential ligands to this site. To our knowledge, this has not been reported for Bfr. Both ligand (heme)-based and fragment-based pharmacophores were generated and employed for screening. Several potential hits were found. In addition to several protoporphyrin IX analogs, several non-heme compounds were also found (Figure 11). These compounds were purchased and screened by dynamic light scattering (DLS) for their effects on the quaternary structure of Bfr in the absence of heme. Several of the compounds exhibited the ability to enhance the amount of 24-mer structure in solution, as measured by DLS. Although further studies on this interesting effect are warranted, these novel effects might be useful in future studies in the application of Bfr to chemotherapy. For example, LB6 is an analog of a compound that blocks NFkB and MAPK activation pathways [123], LB9 is an analog of a set of compounds used to identify potential antagonists of the A3 adenosine receptor [124], and FB5 is an analog in an investigation of CDK2 inhibitors [125]. 

Further molecular design principles can be anticipated from these proof-of-principle findings, which provide a unique nanodimensional tool for biomaterials applications. Future studies will focus on developing alternate cavity affinity strategies as well as utilizing N-terminal fusion peptides and chemical modification to functionalize the Bfr outer surface for targeting applications [32,62,65,126]. 

## Figures and Tables

**Table 1:** N-Terminal peptide fusions on C-terminal His-tagged Bfr ^1^.

Name	Sequence
Qtag1-Bfr	MKETAAAKFERQHMD SPDLH-
Qtag2-Bfr	MKETAAAKFERQHMD SGGGG-
Qtag3-Bfr	MERLQQPT GGG-
Qtag4-Bfr	MERLQQPT GGGG-
SortTag1-Bfr	MLVPR GGGGG-

^1^ Enzyme recognition (red) and linker (green) residues are colored. Underlined glutamines are residues shown from the literature to be the target glutamine for TGase modification.

## Data Availability

Data is contained within the article or Supplementary Materials.

## Supplementary Materials

The following are available online at https://www.mdpi.com/article/10.3390/molecules28124663/s1, Materials: cloning expression and isolation of WT bacterioferritin and His-tag bacterioferritin; heme reconstitution; computational modeling of His-tag Bfr; sample preparation for size exclusion; fluorescence analysis; dynamic light scattering; transmission electron microscopy; heme analog syntheses; mass spectrometry; NTA-dye encapsulation; SF encapsulation; AuNP encapsulation; incorporated modified heme fragmentation by mass spectrometry; incorporated modified heme evaluation; His-tag GFP encapsulation; purification of His-tag GFP; encapsulation and evaluation of His-tag GFP within NTA-heme containing Bfr; transglutaminase (TGase)-catalyzed reactions; N-terminal Qtag and SortaseTag Surface Modifications on His-tag Bfr; figure of ligand-based pharmacophore; figure of ligand-based pharmacophore; DLS results for His-tag Bfr in the presence of protoporphyrin IX analogs; DLS results for His-tag Bfr in the presence of ligand-based pharmacophore selected analogs; DLS results for His-tag Bfr in the presence of fragment-based pharmacophore selected analogs. Two videos: BacterioferritinOverview.mov; HemeOrientation.mov. References [127,128,129,130,131,132,133,134] are cited in the Supplementary Materials.

## Sample Availability

Samples of the genetic constructs are available from the authors upon reasonable request.
