# Supplementary material for: Molecular Engineering of E. coli Bacterioferritin: A Versatile Nanodimensional Protein Cage
Source: Molecules. 2023 Jun 9;28(12):4663. doi: 10.3390/molecules28124663 (PMC10301331; doi:10.3390/molecules28124663)
Supplement: Supplementary file 1 [file molecules-28-04663-s001.zip › molecules-2372338-supplementary.pdf]

SUPPLEMENTARY MATERIALS

Table of Contents

MATERIALS 3

CLONING, EXPRESSION AND ISOLATION OF WILD TYPE (WT) BACTERIOFERRITIN AND HIS-TAG BACTERIOFERRITIN 4

HEME RECONSTITUTION 7

COMPUTATIONAL MODELING OF HIS-TAG BFR 7

SAMPLE PREPARATION FOR SIZE EXCLUSION 8

FLUORESCENCE ANALYSIS 8

DYNAMIC LIGHT SCATTERING 9

TRANSMISSION ELECTRON MICROSCOPY 9

HEME ANALOG SYNTHESES 10

MASS SPECTROMETRY 11

NTA-DYE ENCAPSULATION 11

SF ENCAPSULATION 12

AUNP ENCAPSULATION 12

INCORPORATED MODIFIED HEME FRAGMENTATION BY MASS SPECTROMETRY 13

INCORPORATED MODIFIED HEME EVALUATION 14

HIS-TAG GFP ENCAPSULATION 15

BIS-NTA-HEME SYNTHESIS AND PURIFICATION 15

PURIFICATION OF HIS-TAG GFP 17

ENCAPSULATION AND EVALUATION OF HIS-TAG GFP WITHIN NTA-HEME CONTAINING BFR 17

TRANSGLUTAMINASE (TGASE)-CATALYZED REACTIONS 19

N-TERMINAL QTAG & SORTASETAG SURFACE MODIFICATIONS ON HIS-TAG BFR……………………………………………………………………………...25

Materials

Streptavidin FITC (Life Technologies, Frederick, MD, USA), a fluorescein-labeled tetrameric streptavidin, termed SF in this investigation, dabsyl chloride (Sigma-Aldrich, Bellefonte, Pennsylvania, USA), biotinamidocaproyl-nitrilotriacetic acid (Biotin-X NTA; AnaSpec, Fermont, CA, USA), Black Hole Quencher® 10 (BHQ®-10; Biosearch Technologies, Novato, CA, USA), Pro-Q® Sapphire 365 oligohistidine gel stain (Invitrogen Molecular Probes, Burlington, ON, Canada), and Ni-NTA-Nanogold^{TM} (Nanoprobes, Yaphank, NY, USA; 5 nm gold nanoparticles (AuNP) coated with a polymer displaying nitrilotriacetic acid (NTA) functional groups were utilized in this investigation. The Pro-Q® Sapphire 365 oligohistidine gel stain solution was determined to contain approximately 9.2 μM dye, 60.7 μM Ni^{2+}, 120 mM Na^{+} in 57 mM PIPES buffer (data not shown). The Pro-Q dye has a maximum excitation wavelength at 345 nm and a maximum emission wavelength at 440 nm. The AuNP were provided as a 0.5 μM solution in 50 mM 3-(N-morpholino) propanesulfonic acid (MOPS), pH 7.9) and were used for encapsulation studies. Formvar-Carbon 400 mesh, Cu Grids (Canemco-Marivac, Gore, QC, Canada), Ammonium molybdate ($(NH_4)_6Mo_7O_{24}$) (Sigma-Aldrich, Saint Louis, MO, USA), and uranyl acetate ($UO_2(CH_3COO)_2$) (generously donated by Dale Weber of the Department of Biology at the University of Waterloo) were used in Transmission Electron Microscopy (TEM) experiments. Plasmid pET 22b (Novogen, Mississauga, ON, Canada), and primers (Sigma-Genosys, Oakville, ON, Canada) were used for genetic engineering protocols. Hemin (Sigma-Aldrich, Buchs, Switzerland), PS-carbodiimide resin (Biotage, Hengoed, United Kingdom), N-hydroxysuccinimide (NHS, Sigma-Aldrich, Louis, MO, USA), 5-(aminoacetamido)fluorescein (fluoresceinyl glycine amide; AAF; Molecular Probes® Life Technologies, Eugene, OR, USA), and 7-(diethylamino)coumarin-3-carbohydrazide (DCCH; Molecular Probes® Life

Technologies, Eugene, OR, USA) were used for modified heme synthesis. Hemin (Sigma Aldrich), bis(carboxymethyl)- L–lysine (NTA-amine) (Sigma Aldrich), N-hydroxysuccinimide (Sigma-Aldrich), KP C-18 flash column (Biotage), and P2 resin (Bio Rad) were used for the synthesis of bis-NTA-heme derivatives.

Cloning, Expression and Isolation of Wild type (WT) Bacterioferritin and His-tag Bacterioferritin

The genes coding for *E. coli* WT and C-terminal His-tag Bfr were inserted into the plasmid pET 22b and transformed into BL21(λDE3) cells. The Bfr gene was isolated from genomic *E. coli* DNA by polymerase chain reaction (PCR) and inserted into the *NdeI* and *BamHI* sites of pET22b to generate plasmid pT7bFT. The Bfr gene in pT7bFT was isolated by PCR with the primers in **Supplementary Table S1**, digested with NdeI and XhoI and inserted into pET22b between the *NdeI* and *XhoI* sites. The resulting plasmid, pT7bFT His-tag, codes for Bfr with an in-frame C-terminal His_6 tag after the *XhoI* site. As a result of the addition of the *XhoI* cut site, a Leu and Glu were incorporated at a position preceding the His-tag. (**Supplementary Table S1, Supplementary Table S2**). The plasmid constructions were confirmed by DNA sequencing (Mobix, McMaster University, Hamilton, Ontario). The plasmids were transformed into BL21(λDE3) by $CaCl_2$ transformation. Ampicillin resistant colonies were selected, restreaked and then stored at -80 ^{0}C. The strains BL21(λDE3) containing the plasmid were grown in 5 mL of LB with Ampicillin (50 μg/mL) which were used to inoculate 1.5 L of LB medium with 50 μg/mL ampicillin at 37 °C, shaking at 200 rpm. Optical density was monitored at 600 nm until an absorbance of 0.6-1.0 O. D. units was obtained, indicative of the mid-log phase of growth. Induction of protein expression was initiated with isopropyl β-D-1-thiogalactopyranoside (IPTG) to a final concentration of 1 mM. The culture was shaken at 37 °C

for another 4 hours, and then cells were harvested using centrifugation (Beckmann JA10 centrifuge at 5000 rpm). The cell pellets were collected and resuspended in 20 mL of 50 mM Tris 100 mM NaCl buffer at pH 8.0 such that no clumps were visible, and then passed through an 18-gauge needle. Cells were homogenized using an Emusiflex-C5 (Avestin, Inc., Ottawa, Canada) at a pressure of 17000 psi, and then centrifuged in a Beckmann JA25.5 centrifuge at 15,000 rpm for 20 minutes. The supernatant was collected, heat-treated at 70 °C for 10 minutes and left to cool to room temperature. This solution was centrifuged in a Beckmann JA25.5 centrifuge at 15,000 rpm for 20 minutes to pellet denatured proteins. The supernatant was collected and used in subsequent chromatographic purification steps.

Purification of WT Bfr was performed using an anion exchange resin followed by a size exclusion resin. First, using a Bio Rad Uno™ Q-1 anion exchange resin on a Duoflow chromatography instrument (Bio-Rad Ltd., ON, Canada), WT Bfr was separated from positively charged proteins. The equilibration buffer, 50 mM Tris 100 mM NaCl at pH 8.0, was used to inject 1 mL fractions of crude protein sample onto the column. Bound proteins were eluted using a linear gradient of 50 mM Tris 2M NaCl at pH 8.0. Fractions were analyzed for the presence of WT Bfr using a 15% SDS-PAGE gel, and fractions containing WT Bfr were pooled and concentrated using an Amicon® stirred cell concentrator with a 10000 MW cut off membrane. This concentrated solution was further purified on a Sephacryl™ S-300HR size exclusion resin. The flow rate was 0.5 mL/min and fractions were collected every minute. Again, fractions were analyzed using a 15% SDS-PAGE gel and fractions containing pure WT-Bfr were pooled. The absorbance at 280 and 260 were measured to detect any nucleic acid contamination. If the ratio of 260/280 was above 1.0 then a Bio-Rad type II ceramic hydroxyapatite column was used to separate DNA from the protein sample. In this case, 10 mM phosphate buffer at pH 6.8 was used

as equilibration buffer, and 400 mM phosphate buffer at pH 6.8 was used to elute DNA and protein with a linear gradient. Fractions were examined on a 15% SDS-PAGE gel, and the ratio of 260/280 analyzed to be below 1.0. This sample was determined to have a subunit molecular mass of 18494 Da (predicted: 18495 Da), utilizing a Waters MicroMass Nanospray ESI-QTOF mass spectrometer. The sample was prepared in deionized water using a Nanosep® microcentrifuge spin column with a molecular weight (MW) cut-off of 10000 Da and the sample volume was purged with five times the volume of deionized water. Electrospray ionization (ESI) mass spectrometry data for WT Bfr is shown in **Supplementary Figure S1**.

Alternatively, the DNAse treated WTBfr was precipitated with ammonium sulfate (40% and 80% in 5 mM MgSO4, 1 mM PMSF, 50 mM MOPS-pH 7.5) followed by size exclusion chromatography ((GE Sephacryl™ S-300HR resin in 150 mM NaCl, 50 mM MOPS-pH 7.5) to obtain pure WTBfr fractions.

The absence of heme in the purification buffer allows one to take advantage of the declustered form of bacterioferritin for purification purposes. The His-tag Bfr was purified using a GE immobilized metal affinity chromatography (IMAC) column with Ni^{2+} as the chelated metal. The clarified lysate was loaded onto the column in 50 mM Tris, 100 mM NaCl and 20 mM imidazole at pH 8.0, and the bound protein was eluted with 100% of 50 mM Tris, 100 mM NaCl and 300 mM imidazole at pH 8.0. The eluted fractions were confirmed with a 15% SDS-PAGE gel which revealed pure His-tag Bfr, which required no further purification steps. These fractions were pooled and concentrated on an Amicon® stirred cell concentrator with a molecular weight (MW) cut-off of 10000 Da. The ratio of 260/280 was checked for DNA contamination, but a value higher than 1.0 was never observed. Lastly, the sample was run on a Waters MicroMass nanospray ESI-QTOF mass spectrometer and a subunit molecular mass of

19558.5 Da (predicted: 19560 Da) was determined. This required that the sample be prepared in a solution free of buffer and salt. This was accomplished by buffer exchange in a Nanosep® microcentrifuge spin column, as was done for WT Bfr. ESI mass spectrometry data for His-tag Bfr is shown in **Supplementary Figure S2**.

Heme Reconstitution

His-tag and WT Bfr were reconstituted fully with heme. The purpose of this was to take advantage of several properties of a heme-reconstituted Bfr. The heme acted as a specific reporter group for Bfr with an absorption maximum of 418 nm, and preliminary results indicated an increased equilibrium shift towards the 24-mer state over the dimer and monomer states in the heme-reconstituted protein as shown in the **Supplementary Figure S3**. The insertion method was followed from previous reports on heme reconstitution of Bfr [1]; the general procedure will be outlined here. Hemin was suspended in a solution of 0.1 M NaOH to make a 10 mM solution of hemin. This was diluted with 0.2 M MES buffer at pH 6.5 to a final concentration of 1.5 mM hemin. This solution was added to Bfr such that two equivalents of hemin were provide for each of the 12 heme-binding sites in Bfr. The mixture was held at 80 °C in 0.2 M MES buffer 1 M NaCl at pH 6.5 for 10 minutes, and then left to cool to room temperature. The now fully reconstituted Bfr was run on a Sephadex™ G-25 desalting column with 50 mM Tris, 100 mM NaCl buffer at pH 8.0 to remove any free hemin and MES buffer. The reconstituted heme in Bfr was confirmed via the spectral shift from 380 nm to 418 nm, which was indicative of bis-methionine ligated heme.

Computational Modeling of His-tag Bfr

The 8-mer peptide fusion, Leu-Glu-His-His-His-His-His-His, representing the His-tag with the two amino acid spacer, was added in a random conformation on to the C-terminal Gly residue (residue 158) for each of the 24 protein chains using the *E. coli* bacterioferritin protein structure (PDB:1BFR). This was followed by simulated annealing for each new side chain using the Amber4.1 force field using Sybyl software (Sybyl-X, Tripos Inc. USA). This was performed with 1000 iterations using the Amber4.1 force field with loaded charges and a dielectric constant of 78, with the internal Bfr cavity absent of water molecules. The NBCutoff =8.0 with distance = constant and other parameters were set at default.

Sample Preparation for Size Exclusion

Samples were concentrated, when necessary, with a Pall Nanosep® spin column with a 10 kDa cut off to a volume of 0.5-1 mL before running on a Superose™ 6 or a Sephacryl™ S-300HR resin. Free guest was separated from encapsulated guest using a flow rate of 0.5-1.3 mL/min and collecting fraction volumes of 0.5-1 mL. The running buffer was the same as the dialysis buffer used. The first peak was collected and concentrated to 1 mL before analysis.

Fluorescence Analysis

A Photon Technology International Fluorometer A-1010B Steady-State fluorescence system was utilized in all fluorescence experiments unless otherwise indicated. When necessary, additional components were attached to the Photon Technology International Fluorometer such as a LPS-220B lamp power supply, a SC-500 shutter control, MD-5020 motor driver, and 814 photomultiplier detection system. The encapsulated Pro-Q® Sapphire 365 oligohistidine gel stain samples were analyzed with a fixed excitation wavelength at 345 nm and scanning emission from 400-550 nm. The emission scan for encapsulated SF was recorded over a range of 500-550

nm with a fixed excitation wavelength of 495 nm. Slit widths for incoming and emission light were set to 1 mm.

Dynamic Light Scattering

Particle size measurements were performed on a particle sizer (Zetasizer Nano ZS model ZEN3500, Malvern Instruments Ltd, Worcestershire, England) equipped with a He-Ne laser with 4.0 mW power at a 532 nm wavelength using the size measurement (DLS method) as a software protocol. The scattering light was collected at an angle of 90 ^{o} through fiber optics and converted to an electrical signal by an avalanche photodiode array (APDs). The refraction index was set to 1.450 with 0.001 absorptions. The samples were run in triplicate with the number of runs set to 5 and run duration set to 10 seconds. The particle shape was assumed to be a spherical molecule and can be calculated according to the Stokes-Einstein relationship ($D = kBT/6\pi\eta RH$) as above. For all measurements, dispersant viscosity (η) was set as sample viscosity. The result calculation was in General Purpose mode, which is appropriate for the majority of dispersions and emulsion (Malvern manual, Malvern Instruments Ltd, Worcestershire, England, 2004).

Transmission Electron Microscopy

Transmission electron microscopy (TEM) was performed on a CM10 Philips transmission electron microscope modified with an Advanced Microscopy Techniques image capturing CCD camera. The accelerating potential was set to 100 keV for imaging in bright field mode. Preparation for TEM imaging for both encapsulated SF and the 5 nm gold nanoparticle (GNP) was performed using 400 mesh copper grids with a carbon-formvar coating. First, sample buffer was exchanged for water to prevent the formation of salt crystals as the samples dried onto the grids. Samples were applied to grids by placing the grid upon the sample droplet using ultra-

fine tweezers and incubating for 2 minutes. The grid was removed from the droplet, and excess solution was wicked away using filter paper. The grid was washed subsequently by placing it in a droplet of water and then removing and wicking away excess water. Finally, the grid was placed in 0.5-1% stain solution, excess was wicked away leaving a thin coat of stain solution, which was left covered to dry overnight before imaging.

Heme Analog Syntheses

After the completion of the heme-NHS reaction as described in Methods, excess PS-carbodiimide resin from the reaction was removed by filtration through a funnel packed with glass wool. The filtrate was precipitated by addition into 10 times the volume of chilled isopropanol and centrifuged at 10000 rpm for 10 minutes. The pellet was collected, dried in vacuo, and stored at -20 °C. The proportions of mono- and bis-labeled heme-NHS were analyzed by thin layer chromatography (TLC). The TLC stationary phase used was silica with mobile phase consisting of 9:1 methylene chloride:methanol. The R_f for mono- and bis-labeled hemes were 0.46 and 0.73, respectively in this solvent system.

When purifying the mono-DCCH-heme from the bis-DCCH-heme product, the sample was placed on a Biotage® KP-C18-HS 12 g column which was then washed with 50 mL of 10 mM phosphate buffer pH 7.0, then a gradient from 100% phosphate to 100% acetonitrile (300 mL) was initially utilized, followed by a gradient from 100% acetonitrile to 50% methanol (1240 mL), and subsequently a gradient to a final 100% methanol (100 mL). AAF-heme was purified using a GE Sephadex™ LH-20 resin with a 100 mM phosphate running buffer at pH 8.0. The main reaction products formed under the above reaction conditions were the mono-substituted

dye-heme adducts and hence the purified mono-substituted hemes were utilized in subsequent Bfr reconstitution experiments.

Mass Spectrometry

Samples were analyzed on either a Waters MicroMass® Q-ToF with a nanospray ESI source or a Thermo Scientific Q Exactive™ Orbitrap with an ESI source. Modified heme samples were diluted from a DMF solvent into a 1:1 methanol:water solvent system with 0.1% formic acid. Samples were scanned in positive ion mode. For fragmentation studies, the collisional energies used were between 40-60 eV. Samples run in negative mode were diluted to a final concentration of 1-10 pmole/μL with a solution of 1:1 methanol and water with 0.2% ammonium hydroxide.

NTA-Dye Encapsulation

In order to separate and purify the encapsulated NTA-conjugated Pro-Q® Sapphire 365 dye from the unencapsulated dye, extensive dialysis of the encapsulation solution was followed by size exclusion chromatography (Superose™ 6, 10 mM PIPES buffer (pH 8.0) and 100 mM NaCl or 20 mM Tris(pH 8.0) containing 100 mM NaCl; flow rate 0.5 mL/min). This allowed for the isolation of the multimeric Bfr and the encapsulated guest and for its subsequent analysis (**Supplementary Figure S4**). This analysis included dynamic light scattering which confirmed that a Bfr complex of the correct size was successfully formed in the presence of encapsulated dye molecules, and which maintained associated dye fluorescence even after subsequent dialysis steps to remove non-specifically bound dye molecules. As shown in Methods, this remaining associated fluorescence was entirely dependent upon the presence of His-tag fusion tags present

in engineered Bfr. Evidence for some fluorescence quenching of the encapsulated dye was observed (**Supplementary Figure S5**).

SF Encapsulation

For the Forster resonance energy transfer (FRET) distance calculation, the dipole orientation factor, refractive index of the medium, and the quantum yield were all approximated to 0.667, 1.4, and 0.5, respectively. This calculation was based on a previous model derived for both dabsyl-glutamate and BHQ®10 [2]. Absorbance and emission spectra were collected from the quenchers and SF in the laboratory and used for this predicted FRET distance.

The diameter of the encapsulated SF in Bfr was analyzed with cross sectional intensity plots generated by ImageJ [3] (**Supplementary Figure S6**). In doing this, it was possible to derive diameters for the regions of stain exclusion within Bfr. The processed images had an inner particle size of 3-3.7 nm, which was smaller than expected for the SF guest. Unprocessed images had more comparable sizes to that expected for SF, with inner diameters of 5.4 nm. Other unprocessed images for encapsulated SF exhibited potentially exposed regions. These exposed regions may be a result of the non-native conditions of the TEM imaging. In summary, combined with the size exclusion data, the fluorescence quenching, and the TEM images captured, it was clear that fluorescently-labeled streptavidin was encapsulated within the engineered Bfr.

AuNP Encapsulation

It is of interest to note the approximate number of gold atoms in the 5 nm gold nanoparticle (AuNP) utilized in the present investigation. This helps to highlight the extent of Bfr's capabilities to encapsulate extraordinarily large guests. The number of gold atoms in a 5

nm AuNP can be approximated based on research by Lu, Y. *et al.* [4]. The approximate number of atoms in a 5 nm AuNP, extrapolated from these results, would be 2200 atoms. A separate methodology based on the equation $N_{Au} = 4\pi(R)^3/(3v_g)$, where N_{Au} is the number of atoms, R is the radius, and v_g is the volume of gold, approximated to 17 Å^3, could also be used to approximate the number of gold atoms [5-7]. This analysis yields a value of approximately 3850 gold atoms present in a 5 nm AuNP. Therefore, the estimated number of atoms would be expected to range between approximately 2000-4000 atoms.

Incorporated Modified Heme Fragmentation by Mass Spectrometry

The overall syntheses of the dye-heme analogs are shown in **Supplementary Figure S7**. Synthesized dye-heme analogs were confirmed by mass spectrometry (MS) (**Supplementary Figure S8**). Modified heme incorporated within His-tag Bfr was analyzed using MS/MS. After incorporating the modified mono-substituted heme analogs into His-tag Bfr, excess modified heme was separated from Bfr using a Sephadex™ G-25 desalting column with a buffered running solution of 50 mM Tris and 100 mM NaCl at pH 8.0. This separation of incorporated from non-incorporated fluorescent dye-modified heme ensured that what was detected utilizing mass spectrometry was derived from the incorporated modified heme. When examining incorporated mono-DCCH-heme with MS, an 873 Da ion was observed, which corresponded to the molecular weight for the mono-substituted DCCH-heme (**Supplementary Figure S9**). This ion was fragmented with MS/MS experiments and an ion corresponding to the loss of water was observed, which indicated the presence of a free carboxylic acid, as was expected for the mono substituted heme. In addition, a 557 Da ion was seen. This 557 Da ion was identical to the observed heme fragmentation pattern, which also contained the signature 557 Da fragment

product. This indicated that mono-DCCH-heme remained bound to His-tag Bfr during chromatographic separation. The same experimentation was performed with mono-AAF-heme incorporated within Bfr (**Supplementary Figure S10**). A 1002 Da ion, which matched the expected mass for the mono substituted AAF-heme, was fragmented using MS/MS experiments and ions corresponding to a carboxylic acid cleavage and a 557 Da ion were both observed, as with the heme fragmentation. Again, this analysis suggested that mono-AAF-heme was incorporated within His-tag Bfr.

Studies on the modification of heme propionates in Bfr, to the authors' knowledge, has not been reported previously. However, modified heme cofactors have been incorporated in myoglobin [8, 9]. The results in this publication provide further evidence that heme propionate groups, which extend into the internal cavity of Bfr, can be functionalized and re-inserted into Bfr, expanding the protein's versatility.

Incorporated Modified Heme Evaluation

Modified hemes reconstituted in place of native heme in Bfr exhibited red-shifted Soret bands, indicative of the bis-methionine coordination of heme within Bfr (**Supplementary Figure S11a-c).** Native polyacrylamide gel electrophoresis (PAGE) was used to investigate the presence of the fluorescent marker from the dye-modified heme analogs associated with the 24-mer Bfr protein. The native gels were prepared as 12% acrylamide gels using standard methods and a Bio-Rad Mini-Protean® system was used to pour and run the gels. Gels were run in buffer absent of detergent at 180 V for 40-50 minutes (**Supplementary Figure S11d**).

In addition to the spectral and native PAGE evaluation, fluorescence anisotropy and quenching experiments were performed to confirm that modified heme was properly

incorporation within Bfr (**Supplementary Figure S12).** Anisotropy experiments were performed to compare the rotational freedom of the free fluorophore, the modified heme, and the incorporated modified hemes. When measured, the modified heme incorporated in Bfr had a much higher anisotropy of 0.3 than the free fluorophore, which was 0.034. The free but modified heme showed an anisotropy of 0.059. These results, along with spectral evidence for bis-methionine ligation of the heme analog within Bfr, is entirely consistent with insertion of the heme analog in the heme pocket in Bfr. In addition to these anisotropy measurements, a Perrin plot was used to evaluate the size of the rotating particle that contained the fluorophore. Sucrose was used to change the viscosity of solution from a concentration of 0% to 30% with increments of 3%. The particle diameter of the modified heme incorporated in Bfr was calculated as 10.5 nm, which was close to the actual size of Bfr, that of 12 nm. The free fluorophore, and modified heme were 2.5 nm and 2.7 nm respectively. The size of the free fluorophore itself determined from these calculations was larger than expected based on the actual chemical structure of the fluorophore. This could be explained through resonance energy transfer (RET) [10]. Typically, in Perrin plots of this nature the three lines will intersect at a common point on the y-axes, which is the r_o value [11]. If, as is known with fluorescein, there is RET between fluorophores it will cause a shift in the slope of the Perrin plot. This shift in the slope should cause an overestimation of the true anisotropy value [12]. This effect likely explains why the free fluorophores seemed to be larger than their molecular structure would suggest.

His-tag GFP Encapsulation

Bis-NTA-heme Synthesis and Purification

Protoporphyrin IX (heme) propionate groups were modified with bis(carboxymethyl)-*L*-lysine (NTA) using a carbodiimide based functionalization, as used in previous work. The NHS ester was purified and used as a relatively stable intermediate to react further to generate an amide bond, and introduce novel labels. The formation of the ester was generated using a Biotage® microwave synthesizer, and the reaction was verified with TLC and MS. Based on TLC densitometry results, the relative amount of NHS reaction products for the bis, mono, and free heme were 69.3 %, 30.7 %, and 0 %, respectively. MS was performed using a Thermo Scientific Q exactive orbitrap in positive mode. The bis-NHS-heme was visible as a singly charged ion at 810, and the mono-heme-NHS was observed as a singly charged ion at 713 mass units (data not shown).

The crude product mono and bis NTA-Heme was analyzed with TLC and MS. The MS was performed in negative mode for the highly anionic heme-NTA (**Supplementary Figure S13**). It was observed that both the mono and bis modified form of the NTA-heme were generated. The bis-NTA-heme was observed as the singly and doubly charged ions at 1102 and 550 mass units, respectively. The mono-heme-NTA was observed as a singly charged 858 ion. The first peak from purification on a C-18 column was collected and analyzed with MS and revealed that the ion corresponding to the mono-heme-NTA (858 mass units) had been largely separated from the bis-NTA-heme. The first peak was subjected to further purification using a P2 column to further clean the product. There were two peaks from this column. This first peak was analyzed with MS, and was shown to be the bis-heme-NTA via MS (**Supplementary Figure S14**). The bis product was isolated and dried in vacuo using a Sorval SpeedVac™. The yields of the purification procedures were determined after each purification step. After the first step, the yield was 49.5 % ± 1.2 %, and after the second step the yield was 31.3 % ± 5.9 %.

Purification of His-tag GFP

The sequence of His-tag GFP was verified after transformation into an expression cell line, and can be observed in **Supplementary Table S2**. The expected molecular weight for His-tag GFP was 28111.6 Da, and when the purified His-tag GFP was examined with MS the molecular weight observed was 27959.4 (**Supplementary Figure S15**), which is a 152.2 mass difference. This mass difference may correspond to the loss of methionine from the N-terminus and the additional 18 Da could be due to the dehydration and oxidation steps in the GFP fluorophore formation. The cleavage of N-terminal methionine likely arose from some cleavage of nascent peptide since there was not a distribution of cleaved and un-cleaved protein. Despite this anomaly the purified GFP was used since it maintained its fluorescence characteristics and the His-tag. Sequence alignment of His-tag GFP shows a 99% identity match with green fluorescent protein. The His-tag GFP contained the S65T mutation found in enhanced GFP from the Roger Tsien publication in Nature 373, 1995, as expected.

Thermal stability was measured in terms of the ability of His-tag GFP to retain fluorescence with exposure to higher temperatures for 10 minutes in low and high sodium chloride solutions. The temperatures used were 60, 70 and 80 °C. It was observed that the fluorescence of His-tag GFP at 60 °C for 10 minutes does not result in a loss of fluorescence, whereas both 70 and 80 °C heat treatments for 10 minutes resulted in minor, and total loss of fluorescence, respectively, in both buffer conditions. For the low salt condition, the fluorescent peak of the 70 °C heat treatment was 78% of that of the 60 °C heat treatment. In the high salt condition, the peak of the 70 °C heat treatment was 44% that of the 60 °C heat treatment.

Encapsulation and Evaluation of His-tag GFP within NTA-heme Containing Bfr

A few trial encapsulations of His-tag GFP with WT Bfr were performed by first incorporating bis-heme-NTA into WT Bfr, then adding Ni^{2+} followed by His-tag GFP and mixing at varying temperatures for 10 minutes. Temperatures of 50 °C, 60 °C, and 70 °C were analyzed and then products were separated on a Sephadex S-300 size exclusion resin. The 60 °C condition appeared to give some increased level of encapsulation over controls without heat treatment. This result indicated that the heat treatment appeared to promote more of the His-tag GFP guest to be encapsulated within Bfr containing the bis-NTA-heme. The earlier eluting peak was overlapping exactly where WT Bfr was eluting. Encapsulation trials were further scrutinized under three other experimental criteria: native PAGE fluorescence and staining, fluorescence quenching, and TEM imaging.

The native PAGE studies allowed for evaluation of the intact structure of WT Bfr, and determine if His-tag GFP was associated with the structure. As was seen before staining the gel, under UV excitation, fluorescent bands were present where 24-mer structures of Bfr had previously run. Fluorescence of the free His-tag GFP control was observed to run much further beyond the upper section of the gel. In addition, the WT Bfr with incorporated heme showed no fluorescence. This demonstrated that the fluorescence observed in the samples was not resultant from presence of heme but was the result of the associated His-tag GFP. After staining with Coomassie Blue the protein content was visible and confirmed the presence of Bfr and its association with the fluorescent bands that were present (**Supplementary Figure S16**).

Fluorescent quenching experiments were performed to determine if the encapsulated His-tag GFP was protected excluded from the bulk solution by the protein cage. As previously used, two FRET-based quenching agents, BHQ-10 and dabsyl-glutamate as was done for the fluorescein-labeled streptavidin, as His-tag GFP has a very similar excitation and emission

wavelength profile. This allowed for the use of these same quenchers and useful for comparison. As previously seen, BHQ-10 had stronger quenching effects due to the ability to quench from larger distances. Dabsyl-glutamate had lesser quenching effects, which was again like the fluorescein-streptavidin experiments. Analyses of the controls run in these experiments demonstrated that free His-tag GFP was quenched to a larger extent than the encapsulated His-tag GFP.

Lastly, TEM imaging was used to image encapsulated His-tag GFP. Imaging was conducted as in the previous encapsulation experiments. These images demonstrated that there were some WT Bfr interiors that contained exclusion zones. This was like the images seen with fluorescein-labelled streptavidin. Excluded zones in the interiors of Bfr indicated an inability of the negative stain to penetrate the interior, as opposed to what was seen with the apo-Bfr. This indicated that encapsulated His-tag GFP inside the cavity of Bfr was preventing stain from covering the interior. In addition to the other experimental evidence, these points are consistent with encapsulation of guest His-tag GFP within the protein cage.

Transglutaminase (TGase)-Catalyzed Reactions

A reaction mixture containing *Streptomyces mobaraensis* transglutaminase (TGase)(ZEDIRA GmbH, Darmstadt, Germany; 1 equiv.; 2.66 x 10^{-11} moles), Qtag1-Bfr (226 equiv., 6 x10^{-9} moles), dansylcadaverine (DC) (451 equiv.,1.2 x 10^{-8} moles), and NaCl (38 equiv., 1x10^{-9} moles) in 100 mM Tris-HCl-pH 8, was incubated at different temperatures (from 4 to 50 ℃) for different times (ranging from 1 h to 24 h). The reaction mixture was then loaded onto a desalting column Sephadex™ G10 (GE Healthcare) to exchange the buffer and remove the excess enzyme and crosslinked products. The optimum reaction condition was chosen based on the fluorescence

intensity of the protein band on an unstained SDS-PAGE gel. The gel was further stained with Coomassie Brilliant Blue G-250 dye to visualize for the molecular weight of covalently modified subunit and cross-linked products (**Supplementary Figure S17**). Qtag2-4 were also labelled under these conditions. The emission spectra of these DC-labelled Qtagged His-tag Bfr are shown in **Supplementary Figure S18.** To further confirm that all four Qtags were able to be labelled by the TGase, electrospray mass spectra of the DC-labelled Qtag1-4 His-tagged Bfr proteins are presented in **Supplementary Figure S19.**

The extent of crosslinking and band intensities were used to set optimum reaction conditions for the enzyme to be employed at 10℃ for at least 2 h. Electrospray mass spectrometry was utilized to ascertain the overall mass of the DC-labelled Qtag1-His-tag Bfr protein adduct. The samples for mass spectrometry were run through an IMAC column to separate the modified His-tagged proteins from the excess enzyme. The fractions in the imidazole gradient were pooled, and buffer exchanged into doubly distilled water (DDH$_2$O) deionized water and then injected into the MS instrument (by adding 10 µL of protein to 90 µL of 1:1 MeOH: H_2O with 0.1% formic acid mixture). Alternatively, reaction products were dried using a SpeedVac™ or lyophilized and resuspended in DDH$_2$O for MS analysis.

The DC-labelled Qtag1-His-tag Bfr protein was further treated with cyanogen bromide (CNBr) to assess the position of the DC-modified glutamine. A protein treated with CNBr will be cleaved into fragments at the C-terminal side of methionine residues [13]. There are nine possible fragments from Qtag-Bfr when incubated with cyanogen bromide. The CNBr was suspended in a 70 % formic acid (FA) solution to a final concentration of 50 mg/mL in order to unfold the protein and cleave the protein to the C-terminal side of methionine residues. The reaction mixture was incubated at room temperature overnight, and the excess reagents were

evaporated in an Sorval SpeedVac™. The resulting protein powder was resuspended in DDH2O and buffer-exchanged employing a Sephadex G10™ column (equilibrated with 0.45 µm filtered DDH2O). The fractions containing protein were pooled and analyzed using mass spectrometry. (**Supplementary Figure S20**).

The host-guest AuNP (5 nm)-protein complex (Qtag2-Bfr-Au) was dansylated (DC-Qtag2- Bfr-Au) to illustrate that Bfr can be repurposed on both surfaces while a guest is within the protein shell. TGase-catalyzed reactions (as above) were employed to modify the Qtags with DC. The declustering buffer was then removed by dialyzing the mixture overnight against a reclustering buffer (150 mM NaCl and 25 mM MES-PH 7.4). Dansylation was carried out similarly to that described above. Moreover, the mixture was run on a Sephacryl® S-300 (10/300) size exclusion column using the reclustering buffer to separate the DC-Qtag2-Bfr-Au from the other oligomers in solution. The desired fractions were analyzed on a native-PAGE gel to observe a dansylated 24-mer protein band, while TEM was used to visualize the encapsulated AuNP. (**Supplementary Figure S21**)

N-Terminal Qtag and SortaseTag Surface Modifications on His-tag Bfr

The Qtag1, Qtag2, Qtag3, Qtag4, and SortTag1 engineered versions of the His-Bfr protein were custom gene synthesized by Genscript (Piscataway, USA) and their protein sequences are shown in **Supplementary Table S2**. The commercial plasmids containing these genes were transformed into competent *E. coli* BL21 cells via heat shock for protein expression. Protein isolation was undertaken identically (IMAC chromatography) to the His-Bfr protein which lacked any N-terminal tags.

Investigations on the effects that the various N-terminal tags (Qtags 1-4 and the SortTag1) have on the ability of individual His-Bfr subunits to self-assemble into the α_{24} quaternary structure were undertaken using size exclusion chromatography in the absence and in the presence of heme. Isolated surface tag modified His-tag Bfr proteins were run, as isolated on an Sephacryl TM S-300 HR column with a 1.3 mL/min flow rate (50 mM MOPS- pH 7.4, 0.5 M NaCl) and compared to the proteins after they were pre-incubated with exogenous heme as follows: A solution containing two molar excess of heme (per subunit) was mixed with the surface-tagged Bfr in a high salt buffer (1 M NaCl, 200 mM MES-pH 6.5) and incubated at 70 °C for 30 min or 80 °C for 10 min. This protocol was reported to by Wong et al. to be a satisfactory approach to removing and reinstalling the heme cofactor into Bfr [1]. The high ionic strength ensured that the native structure is restored upon cooling to room temperature, thus making the declustering and reclustering processes reversible. The declustering temperature of 70 °C was used for these experiments since it was found that incubating Bfr at 80 °C for 10 min causes extensive precipitation of the surface tagged Bfr variants (Qtags and SortTag1) but was found not to be a concern for either the HisBfr or WTBfr proteins. To ensure that the subunits are well dissociated to expose the heme binding pockets, the incubation times were extended to 30 min at 70 °C. Centrifugation (to remove excess heme) was performed at a medium speed 5018 *g* to remove excess unreacted heme molecules. The nonspecifically bound heme molecules were further removed by dialysis or by a G-25 desalting column. **Figure 9** (main text) shows the formation of the 24- mer structure from Bfr subunits in the absence and after attempt to reinstall the heme cofactor.

Supplementary Figures

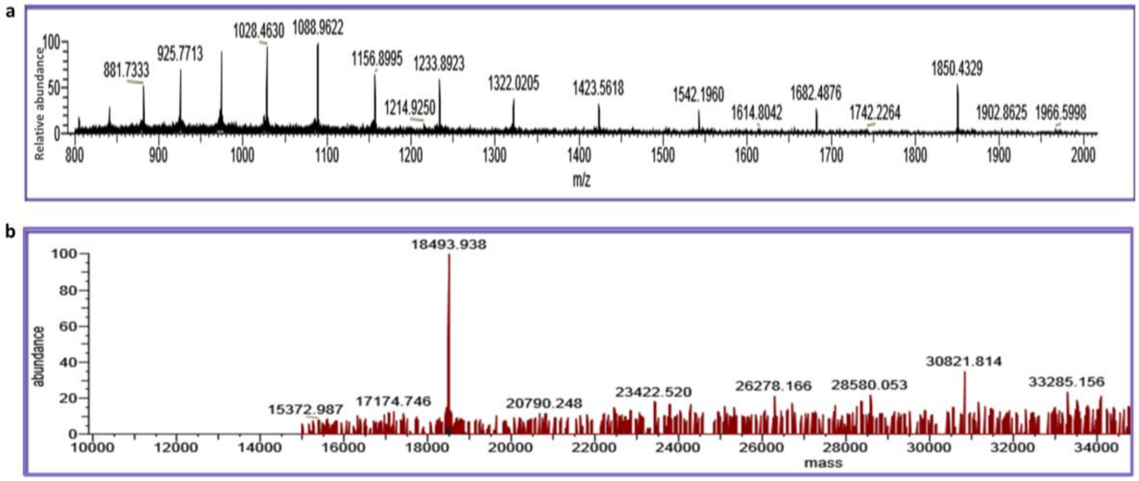

Supplementary Figure S1. Mass spectrum of purified WT Bfr after anion exchange and hydroxyapatite column chromatography. (**a)** The ionization pattern observed for the protein on a nano-spray ESI-QTOF. (**b)** The deconvoluted spectrum of (a), which revealed the correct mass of 18493.9 Da

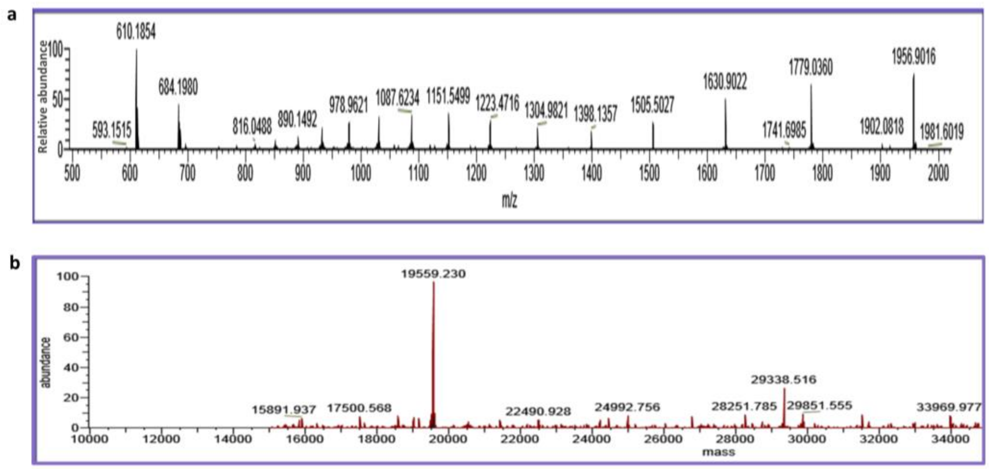

Supplementary Figure S2. Mass spectrum of purified His-tag Bfr post IMAC column chromatography. (**a)** The ionization pattern observed for the protein on a nano-spray ESI-QTOF. (**b)** The deconvoluted spectrum of (a), which revealed the correct mass of 19559 Da

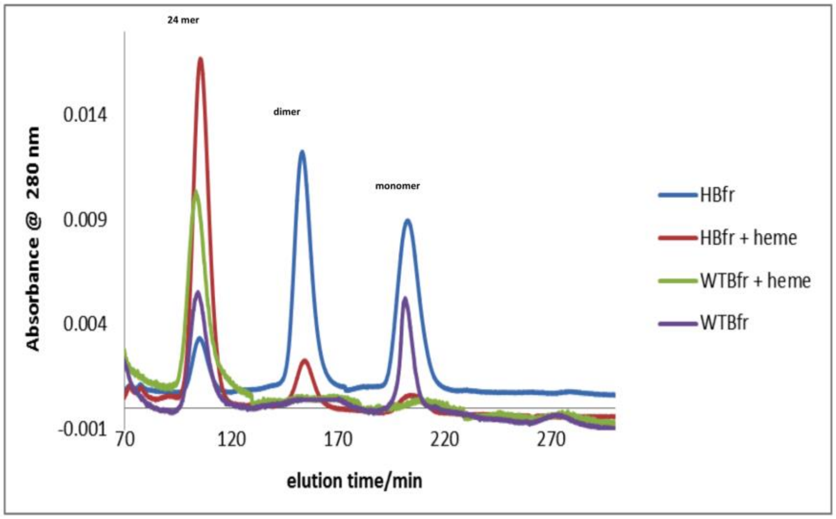

Supplementary Figure S3. Effects of the heme cofactor on the quaternary structure of WT Bfr and His-tag Bfr (HBfr). Size exclusion chromatography was performed on a Sephacryl^{TM} S-300HR (Hi Prep ^{TM} 26/60) resin at 1.3 mL/min flow rate to separate the intact 24-mers from dimers and monomers in solution after the heme reconstitution reaction.

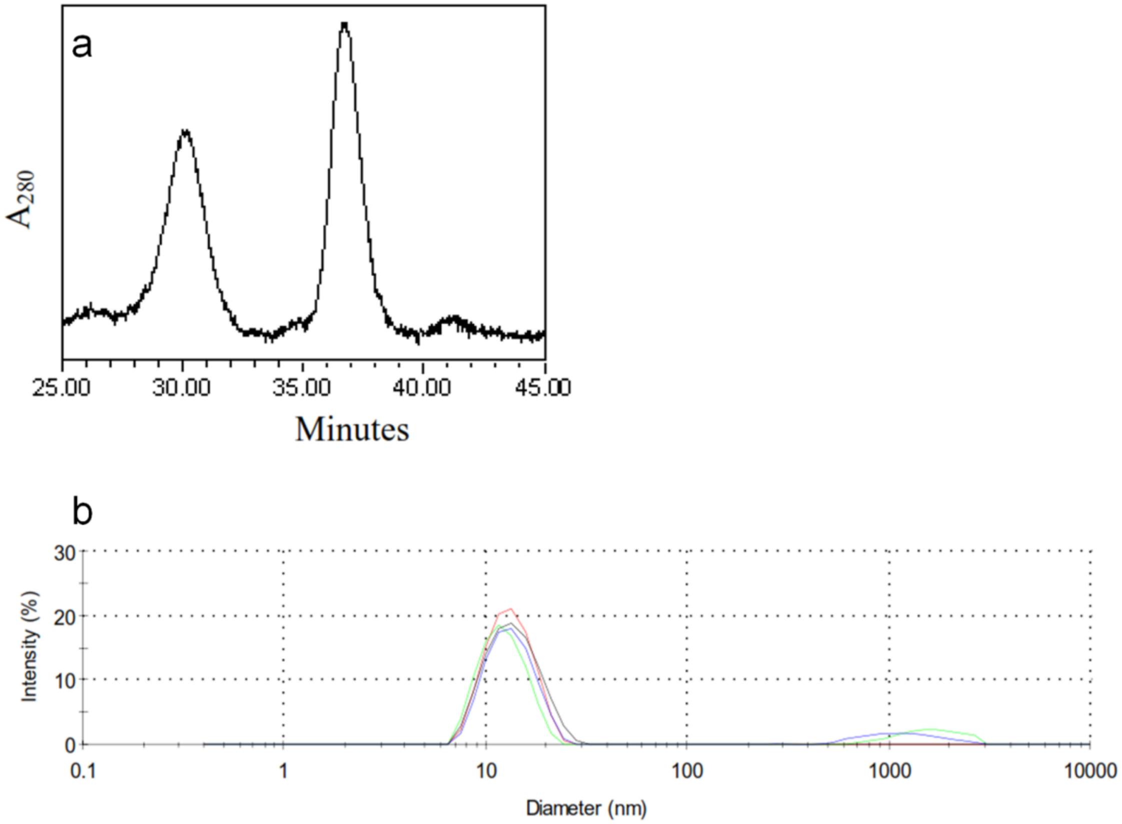

Supplementary Figure S4. Size exclusion chromatography and dynamic light scattering of encapsulated Pro-Q® Sapphire 365 dye in His-tag Bfr. (a) Size exclusion chromatography was performed on a GE Superose™ 6 resin to determine that the intact 24-mer was in solution after encapsulation of the dye. The first peak corresponded to the 24-mer containing the dye, the second peak corresponded to the dimer. (**b)** Correct complex formation was further determined using dynamic light scattering analyses which showed that the protein had the correct exterior diameter of approximately 12 nm.

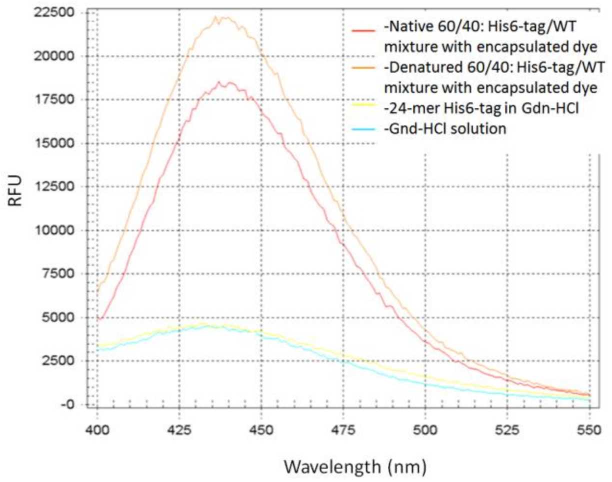

Supplementary Figure S5. Increase in fluorescence of encapsulated Pro-Q® Sapphire 365 dye upon His-tag Bfr host denaturation. The emission spectra were scanned between wavelengths 400-550 nm with a fixed excitation at 345 nm. The samples analyzed were 60/40: His-tag/WT mixture with encapsulated dye, the denatured 60/40: His-tag/WT mixture with encapsulated dye (with 6 M guanidinium hydrochloride (Gdn-HCl) solution), denatured 24-mer His-tag protein lacking encapsulated dye (with 6 M Gdn-HCl), and the 6 M Gdn-HCl solution control.

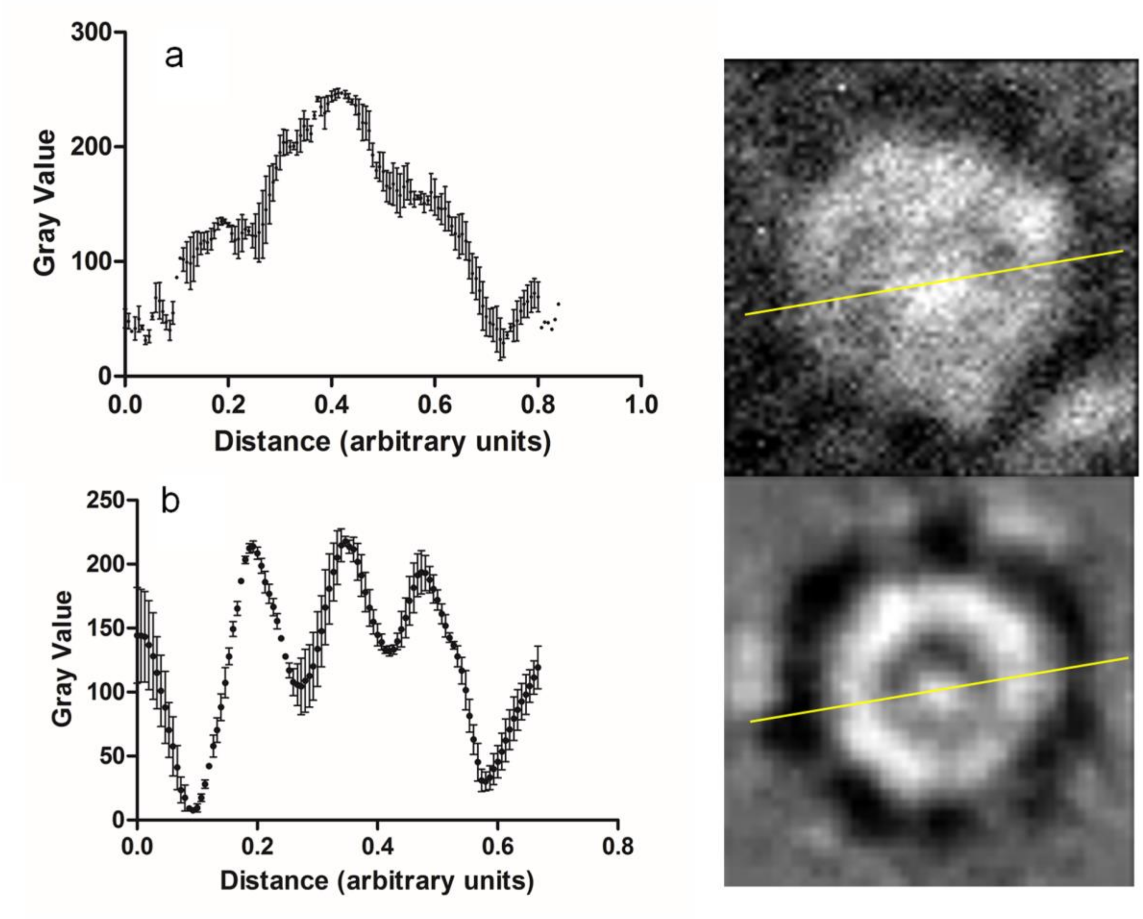

Supplementary Figure S6. Cross sectional examination of the encapsulated SF. (**a**) Analysis of the unprocessed image indicated that the inner particle was approximately 5.4 nm. **(b)** The processed image had an inner particle of approximately 3.7 nm.

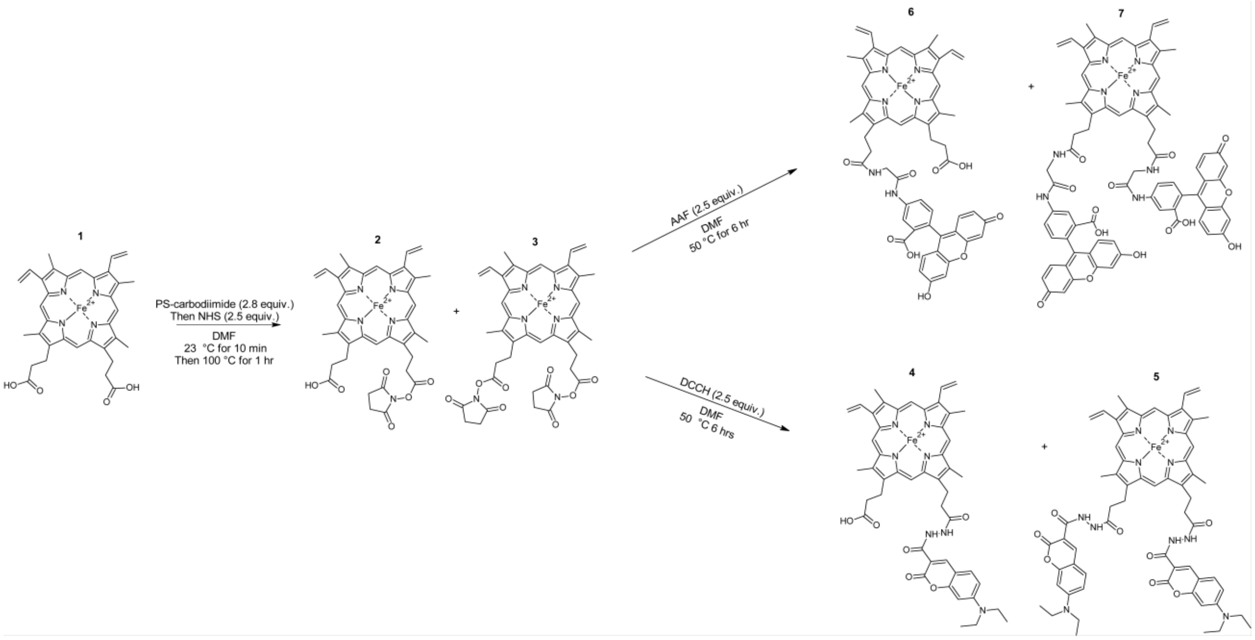

Supplementary Figure S7. Synthetic approach to heme analogs used in this investigation. Mono- and bis-NHS-heme (**2** and **3,** respectively) were synthesized starting from heme (**1**) by activation with PS-carbodiimide resin followed by reaction with N-hydroxysuccinimide (NHS). Mono (**4**) and bis (**5**) DCCH-heme analogs were synthesized from activated NHS-heme using DCCH. Mono- (**6**) and bis- (**7**) AAF-heme analogs were synthesized from activated NHS-heme and AAF.

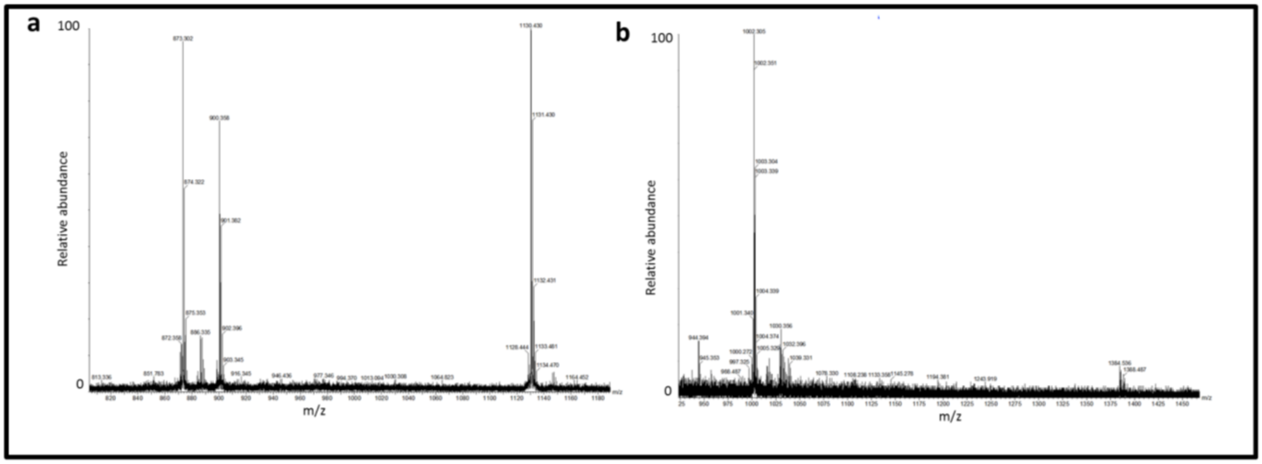

Supplementary Figure S8. Mass spectra of the fluorescent dye-modified heme. (**a)** The synthesized DCCH-heme showed three mass peaks: one mono-substituted DCCH-heme (873 Da), one heme degradation product (900 Da), and one bis-substituted DCCH-heme (1130 Da). The bis-substituted and the heme degradation product were separated from the mono-substituted product before incorporation of the mono-DCCH heme into WT Bfr. (**b)** The synthesized AAF-heme exhibited one major and one minor dye product, the major product was mono-substituted (1002 Da), and the minor peak was the bis substituted hemes (1388 Da).

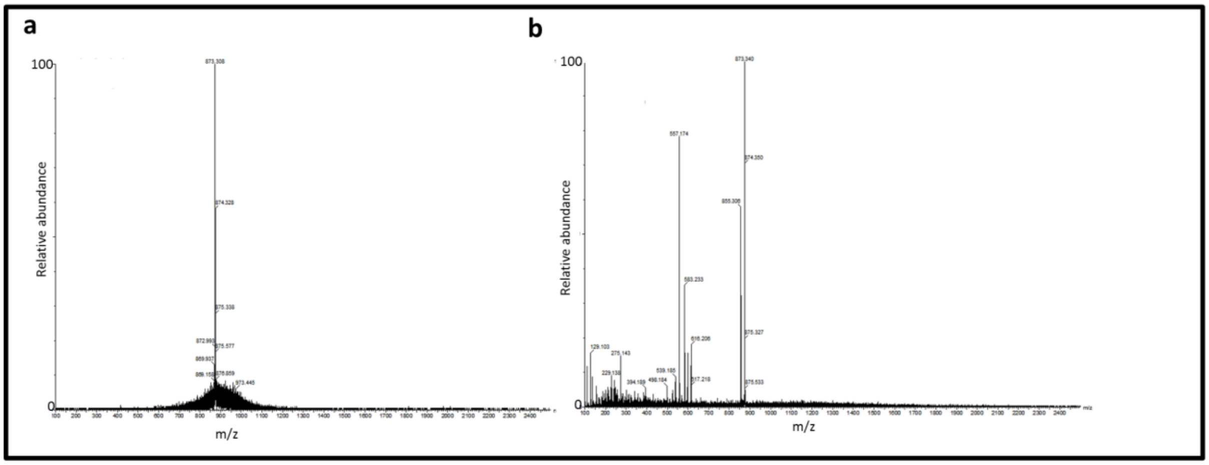

Supplementary Figure S9. Determination of the identity of the incorporated DCCH-heme in His-tag Bfr. **(a)** The detection of the incorporated mono-DCCH-heme molecule in His-tag Bfr as an 873 Da ion. **(b)** This ion was fragmented and formed the ion pattern that was detected previously with free mono-DCCH-heme (Figure S8a). This fragmentation pattern included a 557 Da ion, which was seen with heme fragmentation.

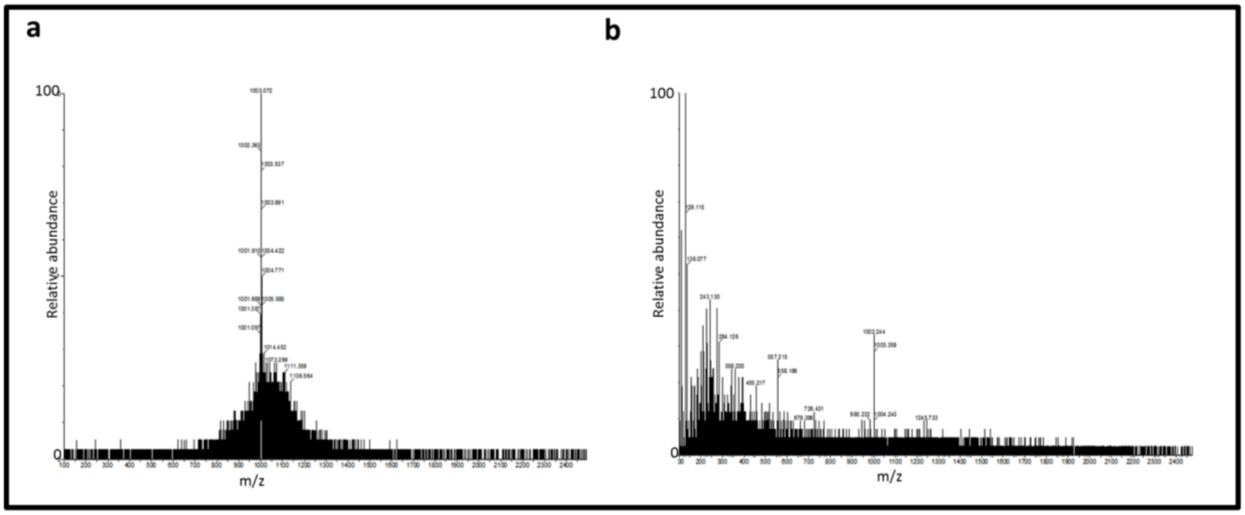

Supplementary Figure S10. Determination of the identity of the incorporated AFF-heme in His-tag Bfr. **(a)** The detection of the mono-AAF-heme molecule (1002 Da ion) after incorporation into Bfr was possible. **(b)** This ion was fragmented and formed the same 557 Da ion observed with heme fragmentation, and a 985 Da ion corresponding to the loss of water from the free carboxylic acid on heme.

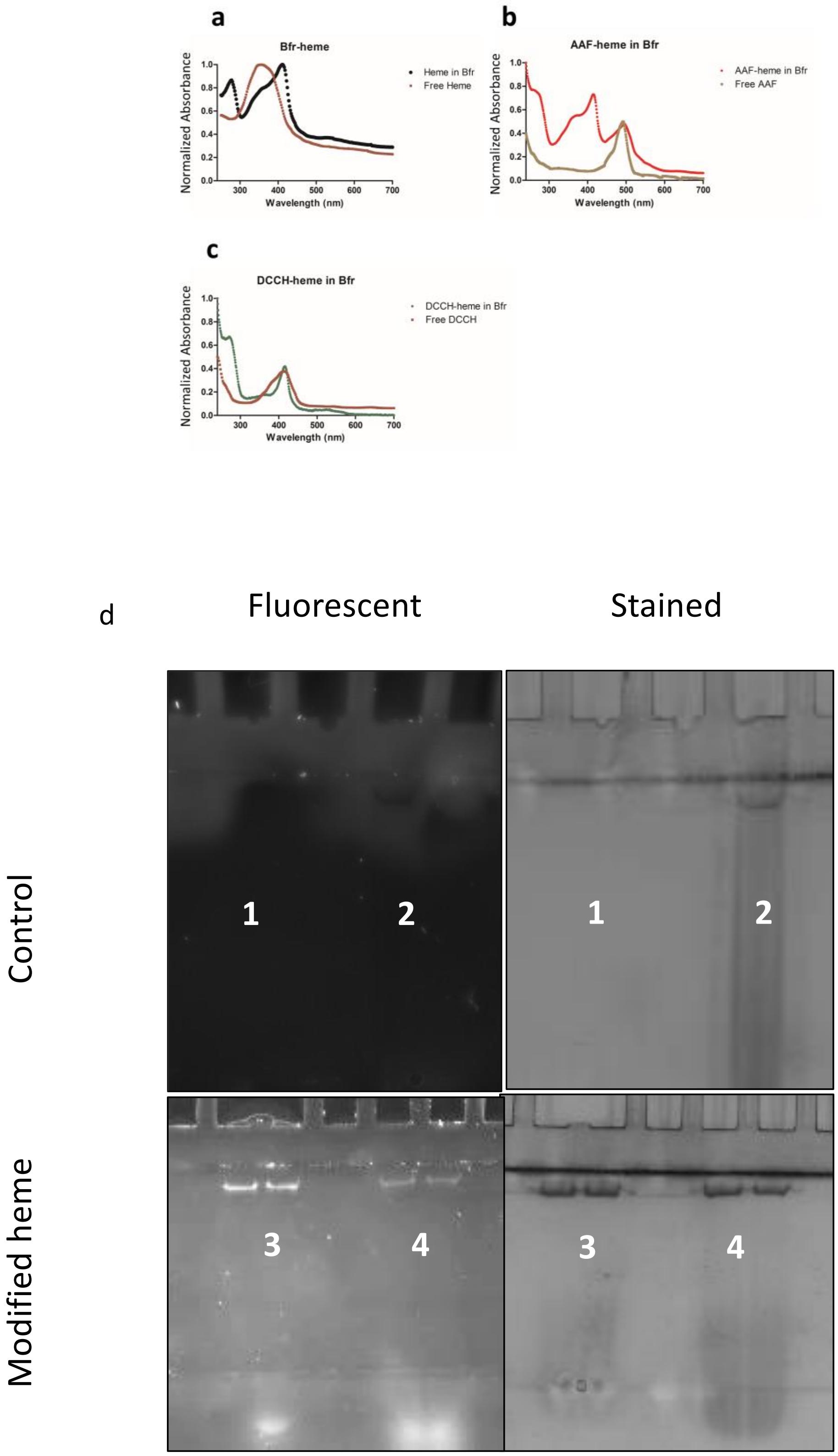
a
Bfr-heme
Normalized Absorbance
Wavelength (nm)
Heme in Bfr
Free Heme
b
AAF-heme in Bfr
Normalized Absorbance
Wavelength (nm)
AAF-heme in Bfr
Free AAF
c
DCCH-heme in Bfr
Normalized Absorbance
Wavelength (nm)
DCCH-heme in Bfr
Free DCCH
d
Fluorescent
Stained
Control
Modified heme
1
2
1
2
3
4
3
4

Supplementary Figure S11. Spectroscopic and native PAGE experiments for the characterization of fluorescent dye-modified heme incorporated in His-tag Bfr. (**a**) Heme, after incorporation into Bfr, underwent an expected spectral shift of the Soret band from 380 to 418 nm when incorporated within Bfr. This same shift was observed for the (**b**) mono-AAF-heme incorporation and the (**c**) mono-DCCH-heme incorporation. In addition to this spectral shift, indicative of bis-methionine coordination, the spectrum also revealed the presence of the AAF and DCCH fluorophores. (**d**) Following incorporation of the modified hemes, the resulting complexes were analyzed using native PAGE to detect fluorescence associated with the non-denatured protein cage. A control gel was run with free mono-AAF-heme (1) and Bfr incorporated with unmodified heme (2). Neither of these samples exhibited fluorescence. Mono-DCCH-heme (3) and mono-AAF-heme (4) incorporated in Bfr were run on a gel and analyzed for fluorescence, which showed fluorescent bands corresponding to the Bfr complex.

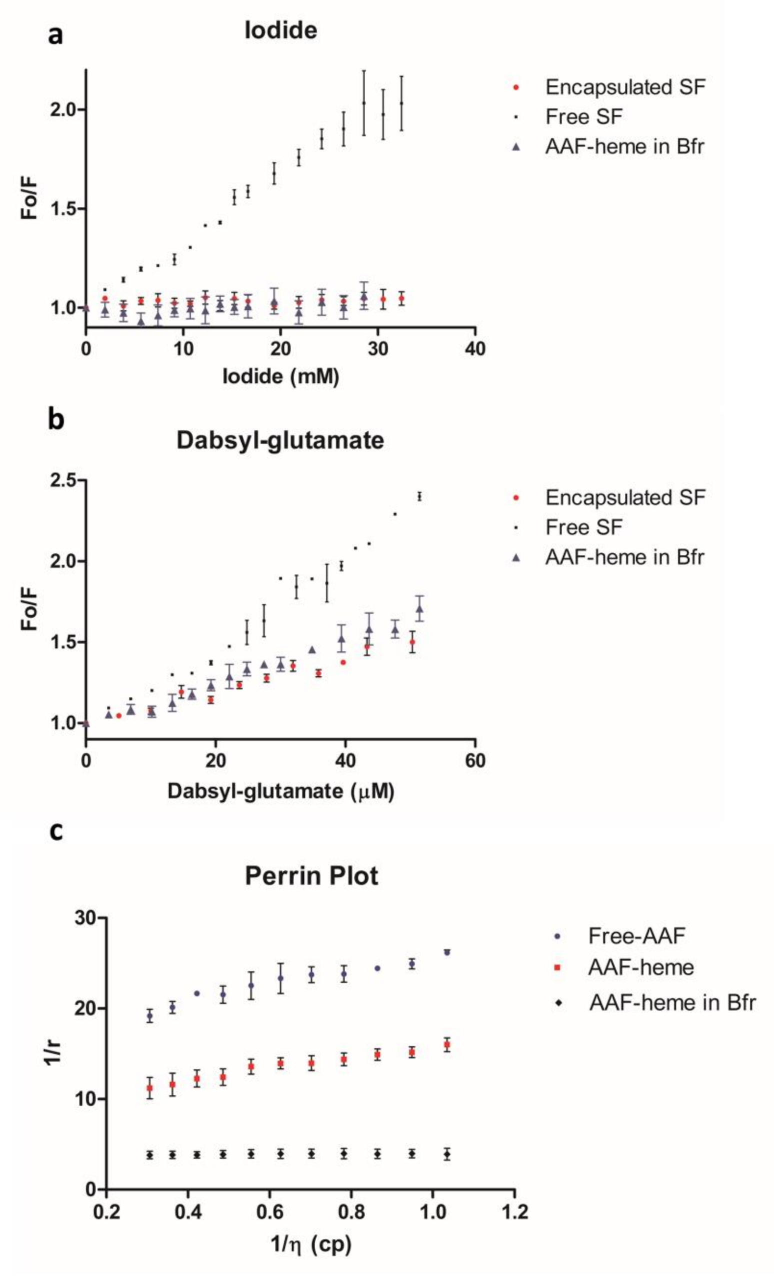
a
Iodide
Fo/F
Iodide (mM)
Encapsulated SF
Free SF
AAF-heme in Bfr
b
Dabsyl-glutamate
Fo/F
Dabsyl-glutamate (μM)
Encapsulated SF
Free SF
AAF-heme in Bfr
c
Perrin Plot
1/r
1/η (cp)
Free-AAF
AAF-heme
AAF-heme in Bfr

Supplementary Figure S12. The AAF-heme incorporated into His-tag Bfr was analyzed employing fluorescence quenching (Stern-Volmer plots) and fluorescence anisotropy (Perrin plots) experiments. The AAF-heme present in reconstituted Bfr was compared with previously studied fluoresceine-labeled streptavidin (SF) in the presence of (**a**) the collisional quencher iodide and (**b**) the FRET quencher dabsyl-glutamate. When compared to the SF fluorescence quenching, AAF-heme behaved similarly to encapsulated SF. (**c**) Fluorescence anisotropy was also analyzed for AAF-heme incorporated into Bfr. It was observed that the fluorophore had a high anisotropy when incorporated into Bfr, whereas the free modified hemes had low fluorescence anisotropies, which indicated that the modified hemes were indeed associated with the Bfr structure.

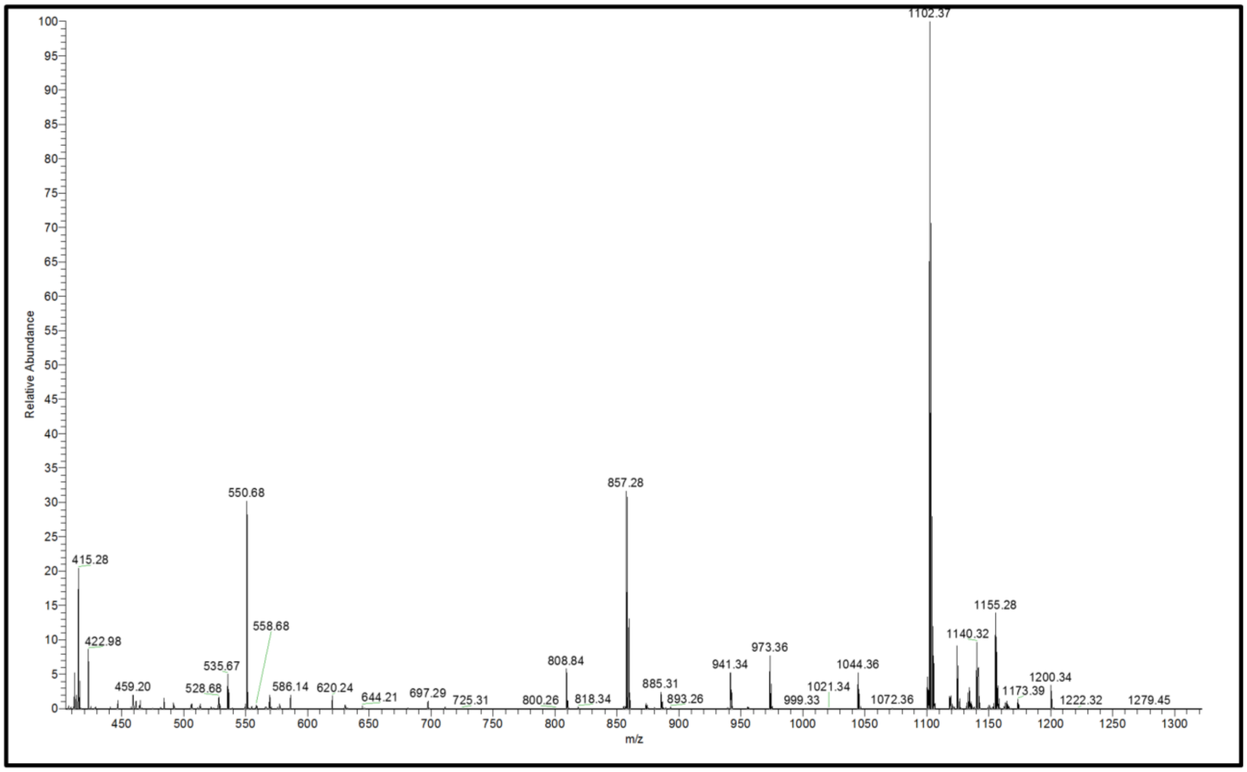

Supplementary Figure S13. Mass spectrum of the synthesized NTA-heme run in negative mode (Thermo Scientific Q-Exactive ESI Orbitrap). The ions at 1102 Da and 550 Da were the singly and doubly charged states of the bis-NTA-heme product. The ion at 857 Da was the mono-NTA-heme product after synthesis.

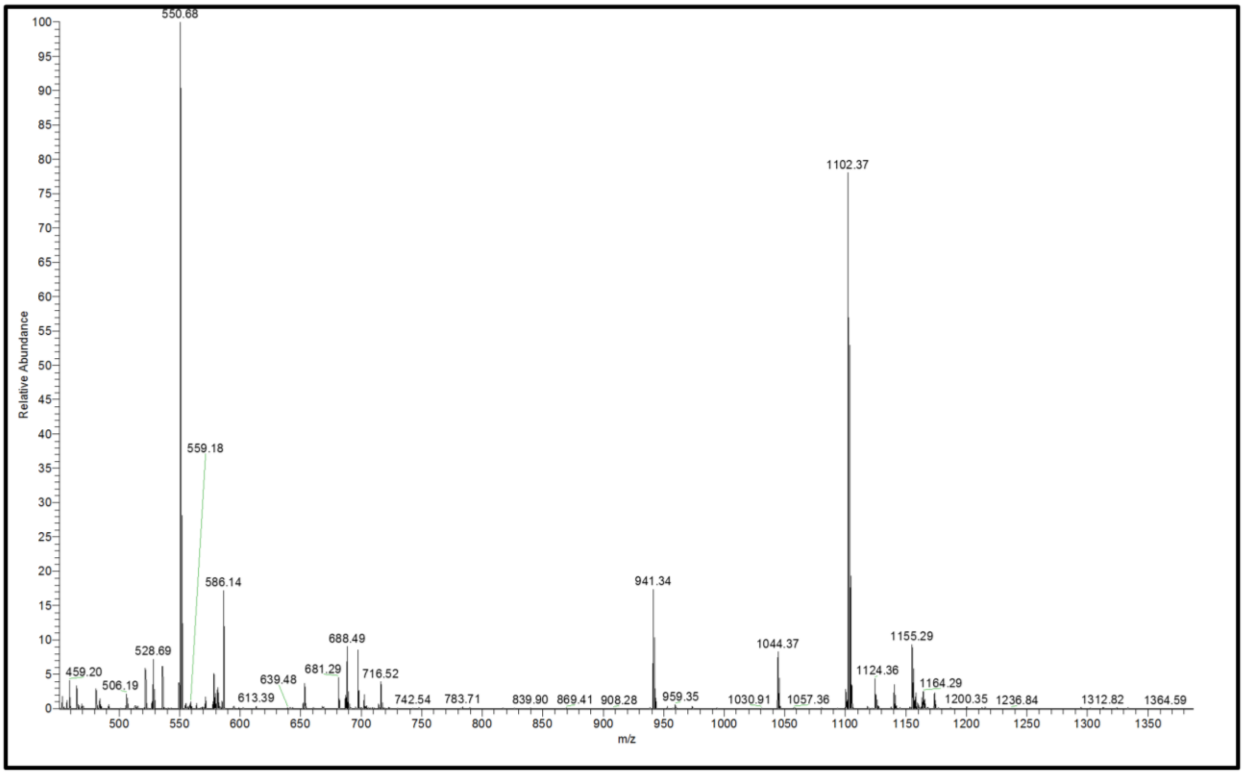

Supplementary Figure S14. Mass spectrum on the purified bis-NTA-heme run in negative ion mode (Thermo Scientific Q-Exactive ESI Orbitrap). The mono-NTA-heme ion at 857 Da is not detectable in the spectrum. Bis-NTA-heme is detectable as its singly and doubly charged ion state at 1102 Da and 550 Da, respectively.

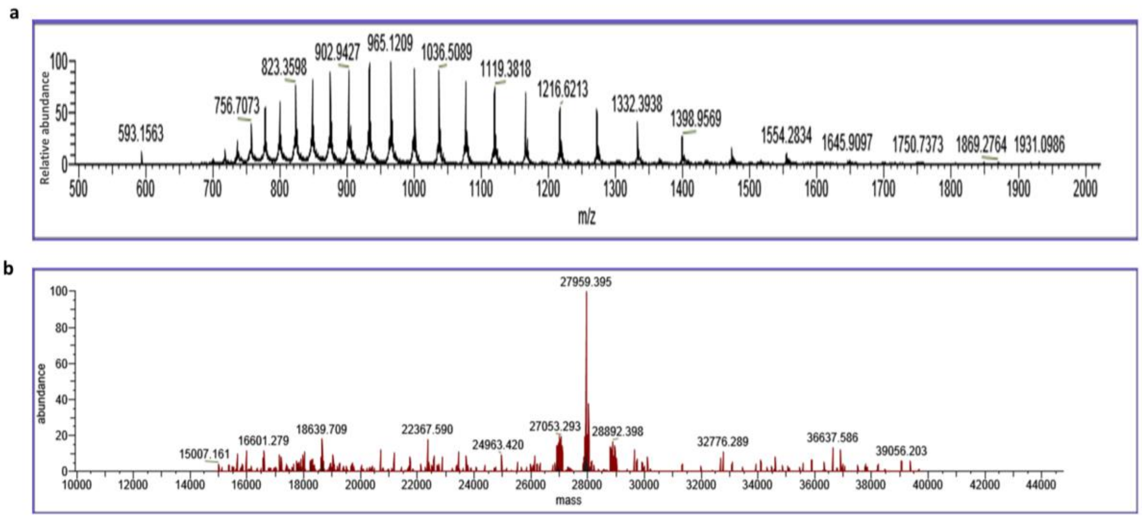

Supplementary Figure S15. Mass spectrum of purified His-tag GFP, post IMAC column chromatograph. (**a**) The ionization pattern observed for the protein on a nano-spray ESI-QTOF.

(**b**) The deconvoluted spectrum of (a), which revealed a mass of 27959.4 Da instead of 28111.6 Da

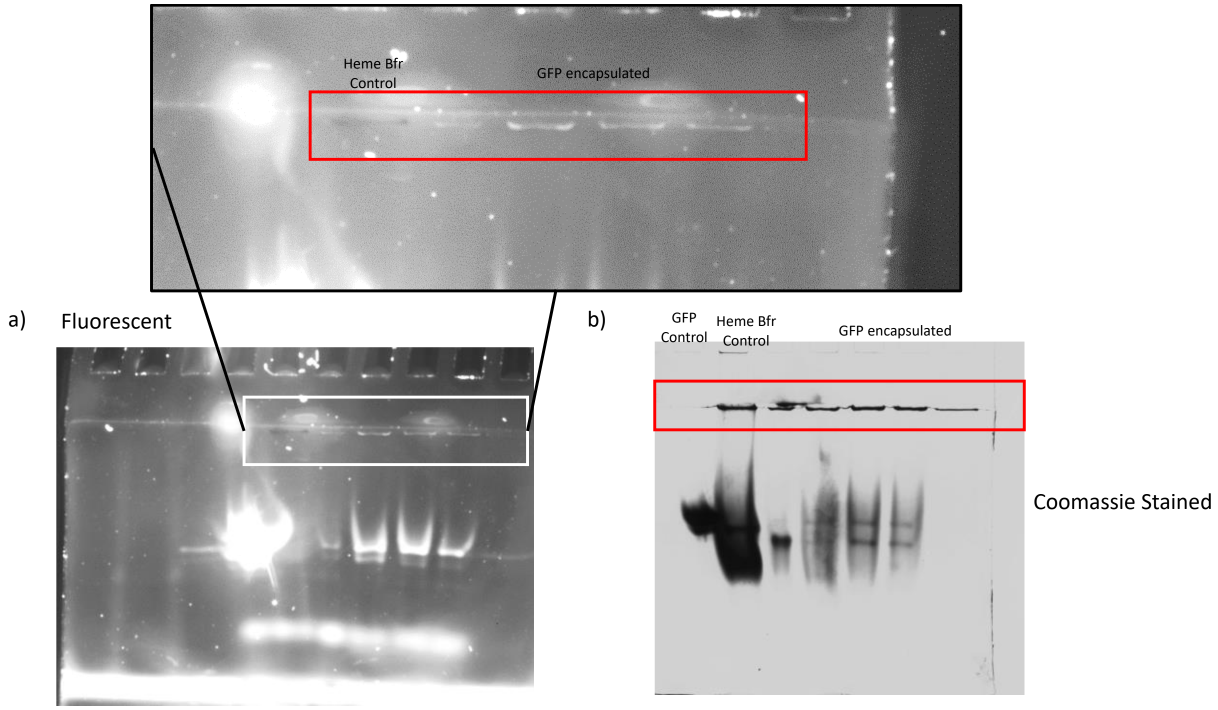

Supplementary Figure S16. Encapsulation trials run on a Native Page Gel. (a) gels were imaged unstained to probe for the presence of fluorescent His-tag GFP. This was visible in the same band as that associated with 24-mer Bfr. The control of Bfr with incorporated heme showed no fluorescence, as seen. **(b)** Staining with Coomassie blue revealed the protein bands

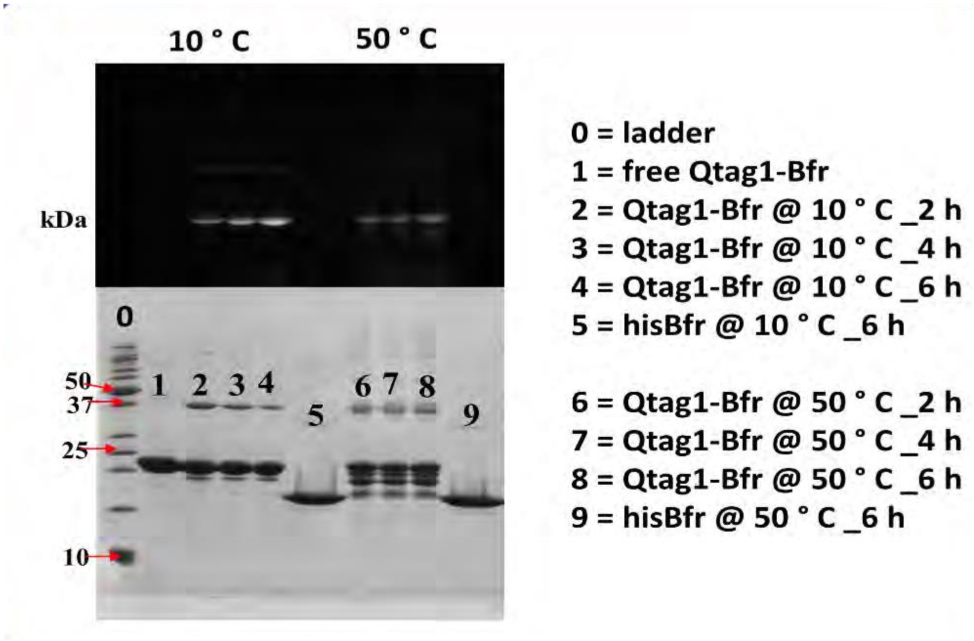

Supplementary Figure S17. SDS-PAGE gel (15 %) showing the modification of Bfr-Qtag1 substrate. The top panel is the fluorescent image of the unstained gel, indicating the presence of dansyl-labeled subunits as well as labeled cross-linked products. The lower panel shows the stained gel with a protein ladder (lane 0, units kDa).

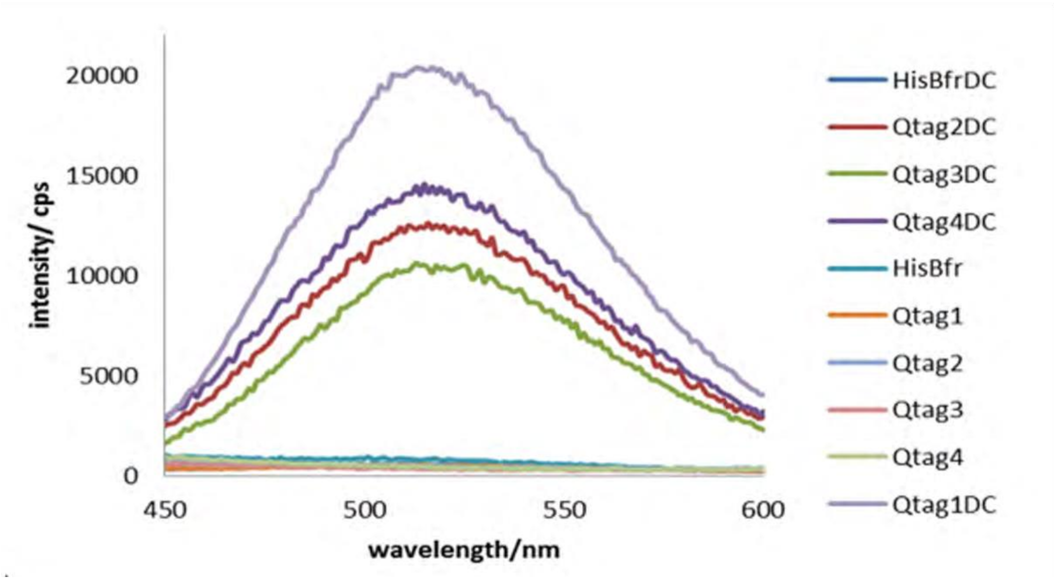

Supplementary Figure S18. Emission spectra of dansylated Qtag-Bfr constructs.

Fluorescent measurements showing all DC labeled constructs except HisBfr are fluorescent (at the dansylcadaverine emission wavelength) when excited at 325 nm, excitation wavelength of dansylcadaverine. Excess DC from the reaction was removed with spin columns before analysis.

Construct	Subunit mass (kDa)	Expected labeled subunit mass (kDa)	Observed labeled subunit mass (kDa)
HisBfr	19560.150	19819.460	19559.170
Qtag1-Bfr	21884.700	22204.160	22202.570
Qtag2-Bfr	21650.000	21969.460	21967.881
Qtag3-Bfr	20715.400	21034.860	21033.041
Qtag4-Bfr	20772.500	21091.960	21090.205

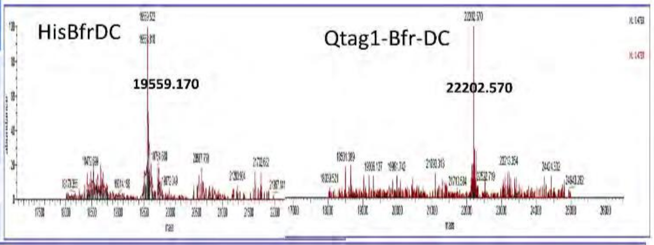

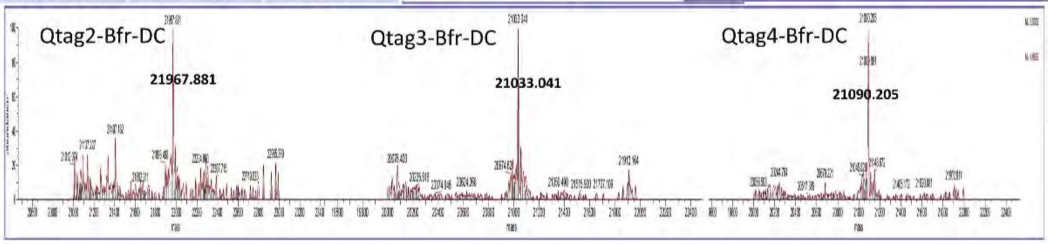

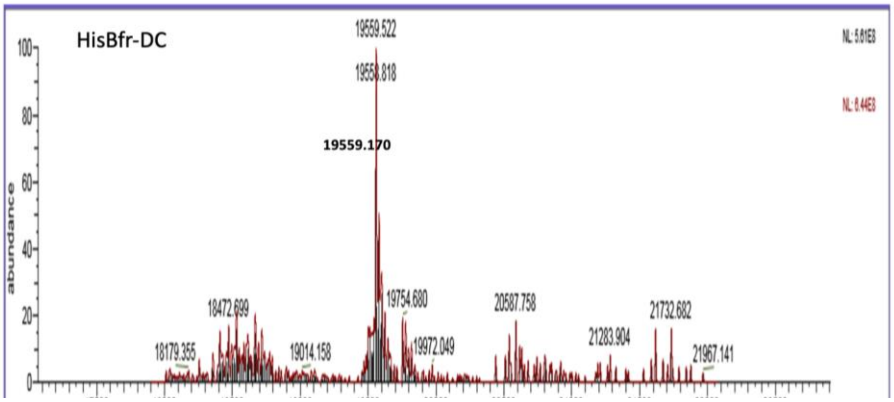

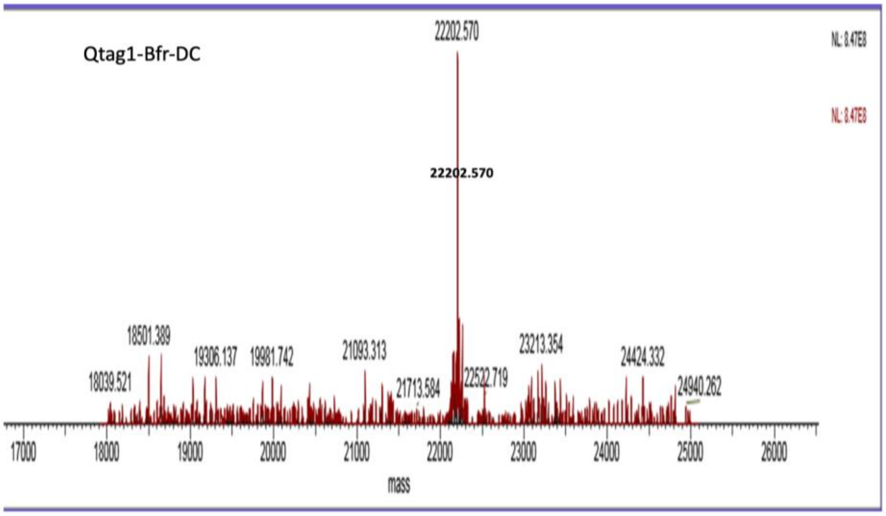

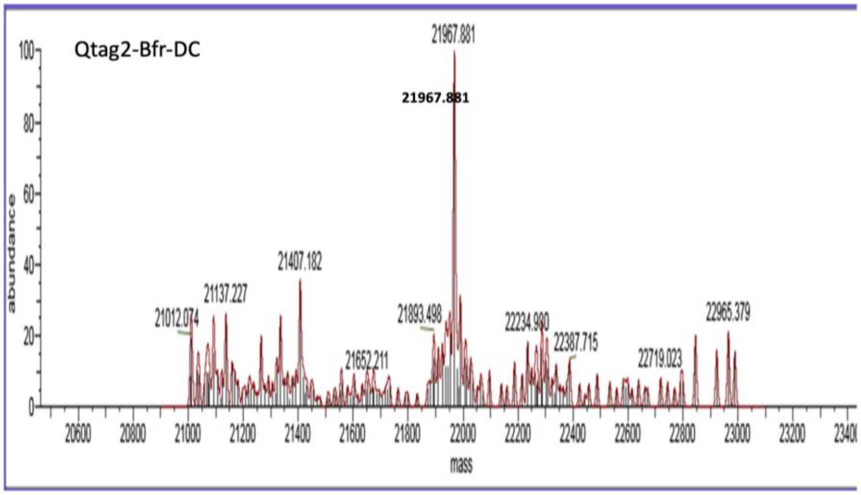
Qtag2-Bfr-DC
21967.881
21967.881
21407.182
21137.227
21012.074
21893.498
22234.980
22387.715
22965.379
21652.211
22719.023
abundance
mass

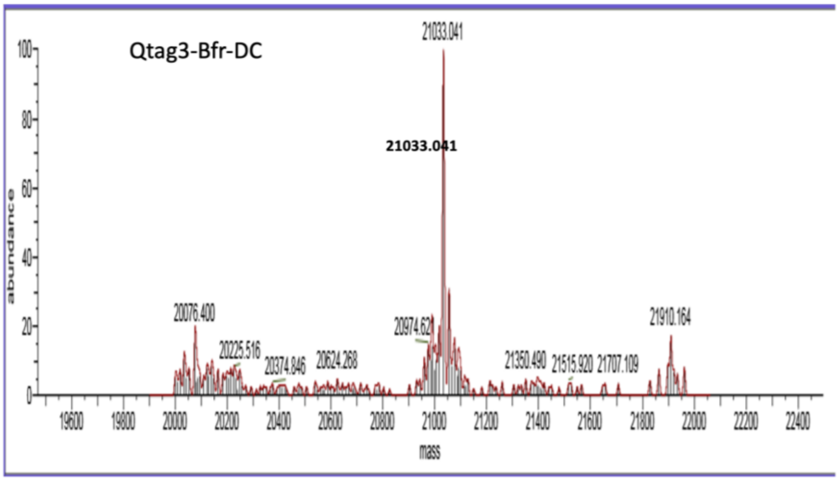
Qtag3-Bfr-DC
21033.041
21033.041
20076.400
20225.516
20374.846
20624.268
20974.621
21350.490
21515.920
21707.109
21910.164
abundance
mass

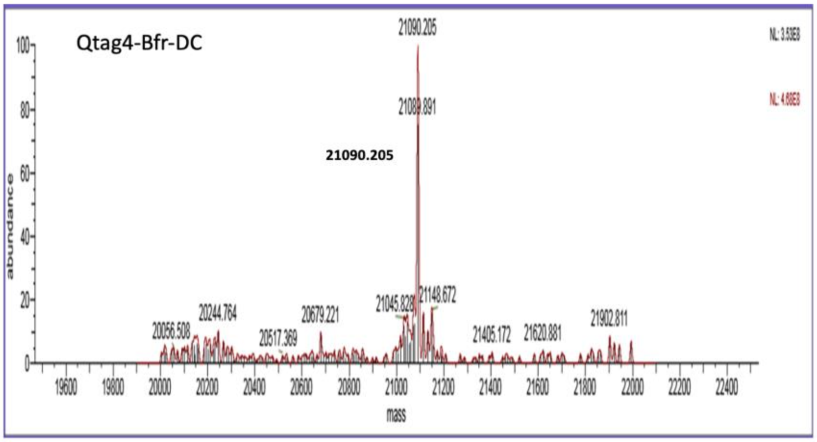
Qtag4-Bfr-DC
21090.205
21089.891
21090.205
20056.508
20244.764
20517.369
20679.221
21045.828
21148.672
21405.172
21620.881
21902.811
abundance
mass

Supplementary Figure S19. Positive ion ESI data for the MTGase catalyzed reactions. The table shows the masses of the unlabeled and DC-labeled subunit and plots of abundance vs. mass for dansylated HisBfr (HisBfr-DC), Qtag1-Bfr(Qtag1-Bfr-DC), Qtag2-Bfr (Qtag2-Bfr-DC), Qtag3-Bfr (Qtag3- Bfr-DC), and Qtag4-Bfr (Qtag4-Bfr-DC). Individual expanded spectra are also presented. HisBfr is not dansylated as the mass for this possible product is not observed.

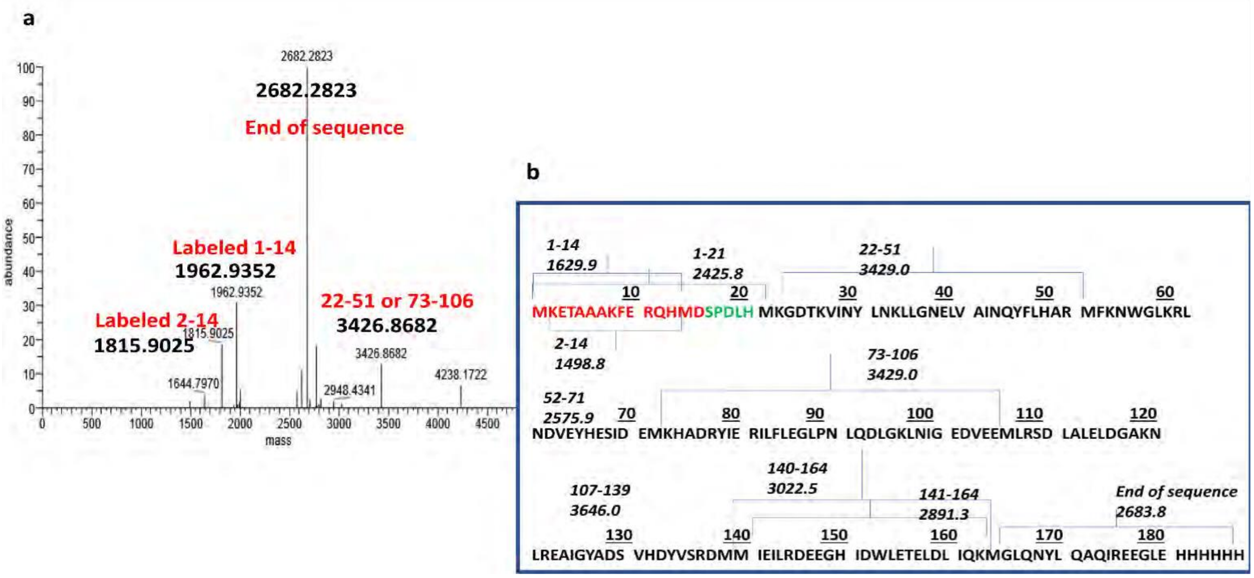

Supplementary Figure S20. Cyanogen bromide fragments of DC-labelled Qtag1-His-tag Bfr. (a) +ESI-MS results showing the end of sequence mass as the most abundant peak and three fragments from the N-terminus. (b) The nine estimated fragments obtained from the ExPASy peptide cutter tool.

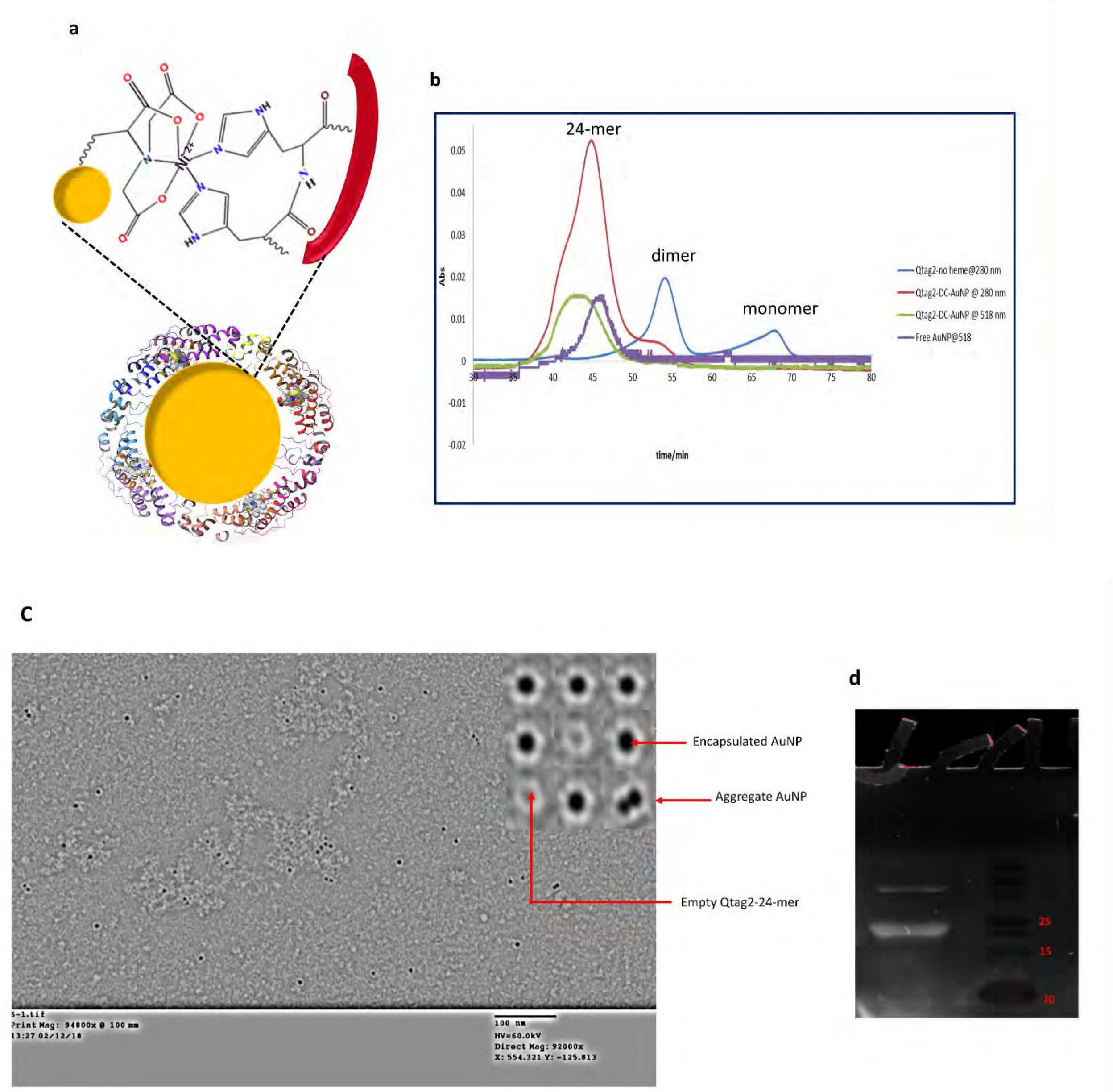

Supplementary Figure S21. External and internal surface modification of Qtag2-His-tag Bfr. Panel a shows a schematic diagram of the His-Ni^{2+}-NTA interaction that likely occurs between the encapsulated NTA-coated AuNP and the His-tag of the cage. (b) SEC elution profile with the encapsulated NTA-coated AuNP (purple) corresponding to an intact 24-mer structure in size. (c) The TEM image of the pooled fractions corresponding to the 24-mer

structure from SEC, indicating successful AuNP encapsulation (d) The SDS-PAGE gel illuminated to show the fluorescence of DC-labeled AuNP@Qtag2-His-tag Bfr, indicating that the AuNP@Bfr has been dansylated on the exterior surface. Molecular weight standards are shown on the right.

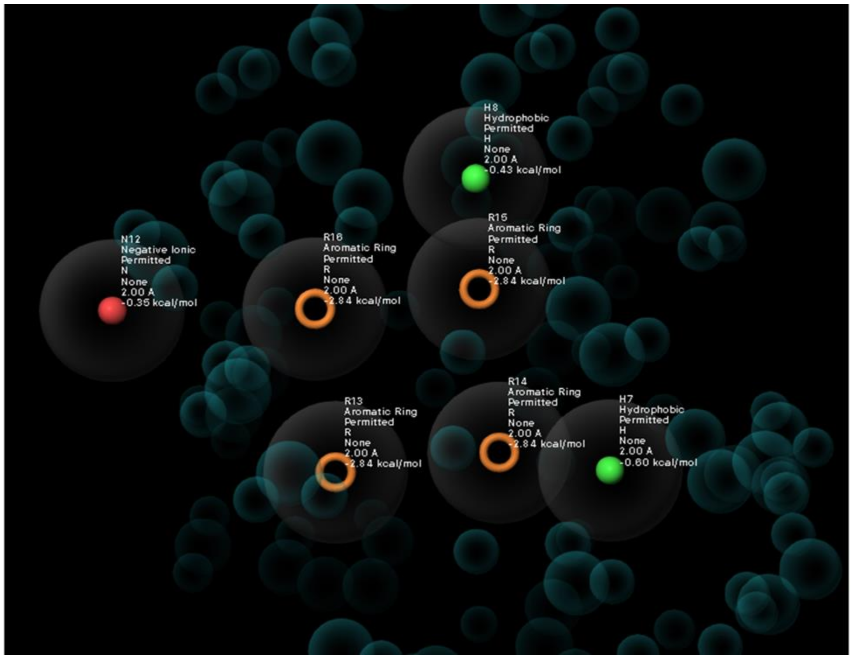

Supplementary Figure S22. The ligand-based pharmacophore generated for the heme binding site in Bfr. The teal spheres represent the excluded volumes, indicating that a ligand could experience steric clashes (from receptor molecules) within these regions. The orange color represents aromatic groups, red represents ionic groups and green represents hydrophobic groups.

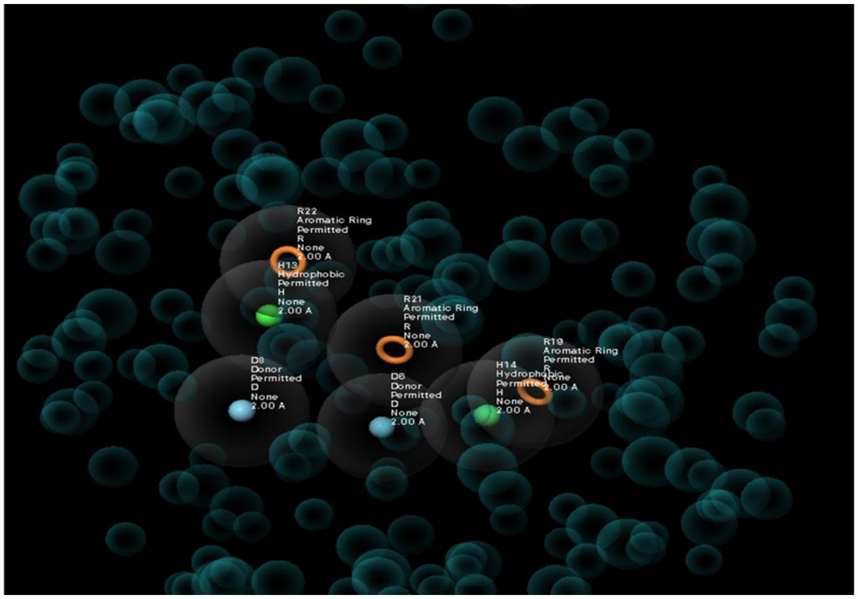

Supplementary Figure S23. The fragment-based pharmacophore generated for the heme binding site in Bfr. The teal spheres represent the excluded volumes, indicating that a ligand could experience steric clashes (from receptor molecules) within these regions. Seven key subsites are shown: aromatic sites (orange), donor sites (light blue), and hydrophobic sites (green).

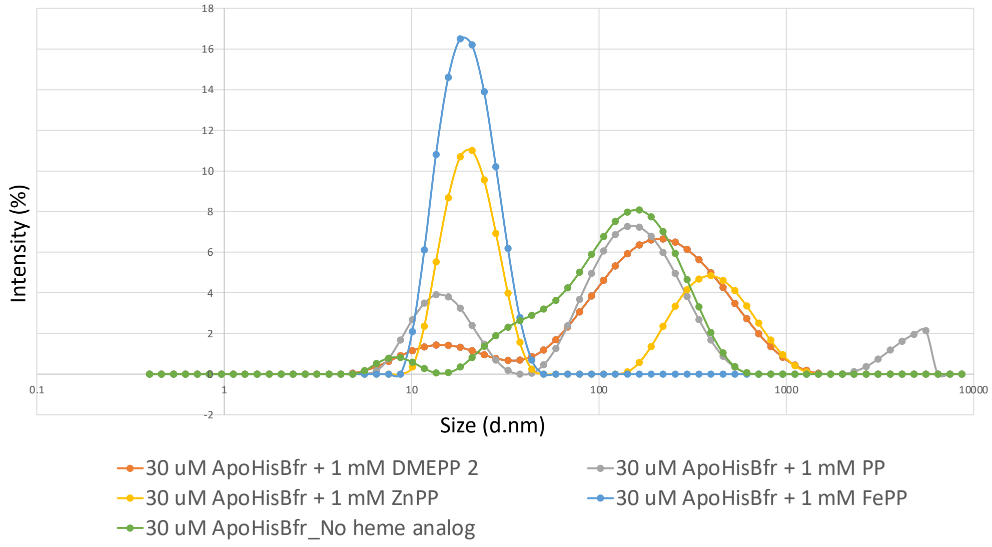

Supplementary Figure S24. DLS results for His-tag BFr in the presence of protoporphyrin IX analogs

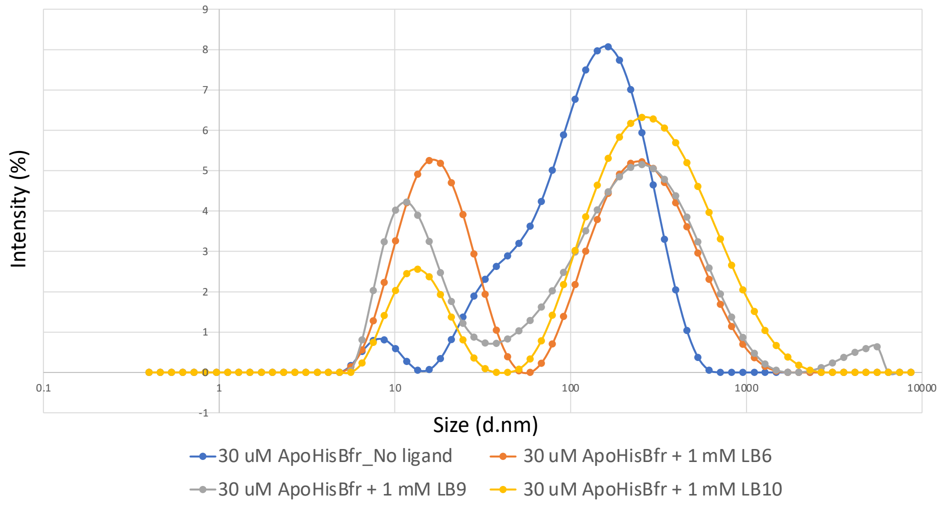

Supplementary Figure S25. DLS results for His-tag BFr in the presence of ligand-based pharmacophore selected analogs

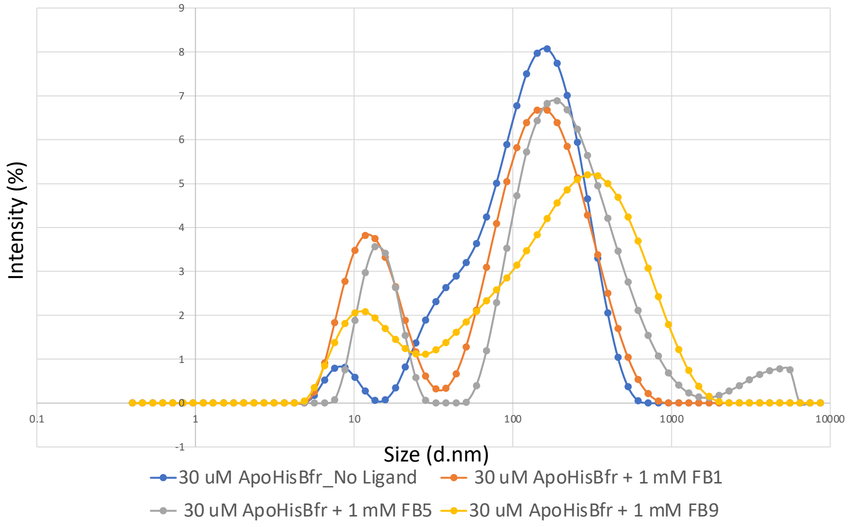

Supplementary Figure S26. DLS results for His-tag BFr in the presence of fragment-based pharmacophore selected analogs

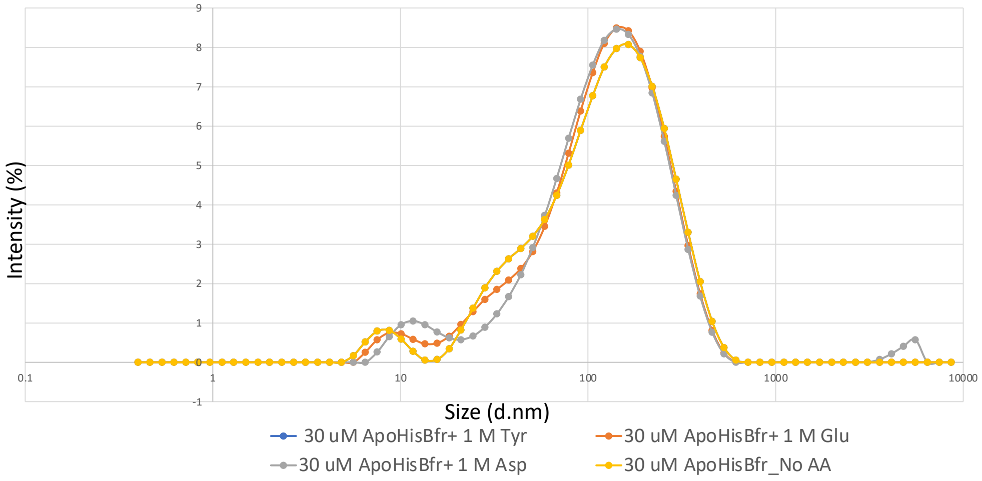

Supplementary Figure S27. DLS results for His-tag BFr in the presence of Tyr, Asp, or Glu

Supplementary Table S1: The forward and reverse primers used to generate bFT-WT with a NdeI cut site at the 5' end and a XhoI cutting site at the 3' end.

Primers	Sequence (5' to 3')	Melting Temperature (T_m) °C
Bfr NdeI Forward	CCAGGATC**CATATG**AAAGGTGATACTAAAG‡	59
Bfr XhoI Reverse	CCG**CTCGAG**ACCTTCTTCGCG‡	60

‡The bold letters represent digestion sites.

Supplementary Table S2: Amino Acid Sequences of the WT and His-tag Bfr. Bold lettering is the attached His-tag with the LE dipeptide between the C-terminal of the wild-type protein and the His-tag. Red colored residues are the "Tags" and the green colored residues are the linker amino acids

WT Bfr	MKGDTKVINYLNKLLGNELVAINQYFLHARMFKNWGLKR<br>LNDVEYHESIDEMKHADRYIERILFLEGLPNLQDLGKLNIGEDVEEMLRSDLALELDGAK<br>NLREAIGYADSVHDYVSRDMMIEILRDEEGHIDWLETELDLIQKMGLQNYLQAQIREEG
His-tag Bfr	MKGDTKVINYLNKLLGNELVAINQYFLHARMFKNWGLKRLNDVEYHESIDEMKHADRYIERI<br>LFLEGLPNLQDLGKLNIGEDVEEMLRSDLALELDGAKNLREAIGYADSVHDYVSRDMMIEILR<br>DEEGHIDWLETELDLIQKMGLQNYLQAQIREEG**LEHHHHHH**
His-tag GFP	MAS**HHHHHH**MVSKGEELFTGVVPILVELDGDVNGHKFSVSGEGEGDATYGKLTLKFICTTG<br>KLPVPWPTLVTTFTYGVQCFSRYPDHMKQHDFFKSAMPEGYVQERTIFFKDDGNYKTRAEV<br>KFEGDTLVNRIELKGIDFKEDGNILGHKLEYNYNSHNVYIMADKQKNGIKVNFKIRHNIEDGS<br>VQLADHYQQNTPIGDGPVLLPDNHYLSTQSALSKDPNEKRDHMVLLEFVTAAGITHGMDELYK
Qtag1 His-Bfr	**MKETAAAKFERQHMD** **S P D L H** M K G D T K V I N Y L N K L L G N E L V<br>AINQYFLHARMFKNWGLKRLNDVEYHESIDEMKHADRYIERILFLEGLPNLQDLGKLNIGEDVEEMLRSDLALE<br>LDGAKNLREAIGYADSVHDYVSRDMMIEILRDEEGHI DWLETELDLIQKMGLQNYLQAQIREEG**LEHHHHHH**
Qtag2 His-Bfr	**MKETAAAKFERQHMD** **S G G G G** M K G D T K V I N Y L N K L L G N E L V<br>AINQYFLHARMFKNWGLKRLNDVEYHESIDEMKHADRYIERILFLEGLPNLQDLGKLNIGEDVEEMLRSDLALE<br>LDGAKNLREAIGYADSVHDYVSRDMMIEILRDEEGHI DWLETELDLIQKMGLQNYLQAQIREEG**LEHHHHHH**
Qtag3 His-Bfr	**MERLQQPT** **G G G** M K G D T K V I N Y L N K L L G N E L V A I N Q Y F L<br>HARMFKNWGLKRLNDVEYHESIDEMKHADRYIERILFLEGLPNLQDLGKLNIGEDVEEMLRSDLALELDGAKNL<br>REAIGYADSVHDYVSRDMMIEILRDEEGHIDWLETEL DLIQKMGLQNYLQAQIREEG**LEHHHHHH**
Qtag4 His-Bfr	**MERLQQPT** **G G G G** M K G D T K V I N Y L N K L L G N E L V A I N Q Y F<br>LHARMFKNWGLKRLNDVEYHESIDEMKHADRYIERILFLEGLPNLQDLGKLNIGEDVEEMLRSDLALELDGAKN<br>LREAIGYADSVHDYVSRDMMIEILRDEEGHIDWLETE DLIQKMGLQNYLQAQIREEG**LEHHHHHH**
SortTag1 His-Bfr	**MLVPR** **G G G G G** M K G D T K V I N Y L N K L L G N E L V A I N Q Y F L<br>HARMFKNWGLKRLNDVEYHESIDEMKHADRYIERILFLEGLPNLQDLGKLNIGEDVEEMLRSDLALELDGAKNL<br>REAIGYADSVHDYVSRDMMIEILRDEEGHIDWLETEL DLIQKMGLQNYLQAQIREEG**LEHHHHHH**

References

1. Wong, S. G.; Abdulqadir, R.; Le Brun, N. E.; Moore, G. R.; Mauk, A. G., Fe-haem bound to Escherichia coli bacterioferritin accelerates iron core formation by an electron transfer mechanism. *Biochem. J.* **2012,** 444, (3), 553-60.
2. Hink, M.; Visser, N.; Borst, J.; van Hoek, A.; Visser, A., Practical Use of Corrected Fluorescence Excitation and Emission Spectra of Fluorescent Proteins in Forster Resonance Energy Transfer (FRET) Studies. *J. Fluoresc.* **2003,** 13, 185-188.
3. Schneider, C. A.; Rasband, W. S.; Eliceiri, K. W., NIH Image to ImageJ: 25 years of image analysis. *Nat. Methods* **2012,** 9, (7), 671-5.
4. Lu, Y.; Wang, L.; Chen, D.; Wang, G., Determination of the concentration and the average number of gold atoms in a gold nanoparticle by osmotic pressure. *Langmuir* **2012,** 28, (25), 9282-7.
5. Leff, D. V.; Ohara, P. C.; Heath, J. R.; Gelbart, W. M., Thermodynamic Control of Gold Nanocrystal Size - Experiment and Theory. *J. Phys. Chem. B* **1995,** 99, (18), 7036-7041.
6. Labande, A.; Ruiz, J.; Astruc, D., Supramolecular gold nanoparticles for the redox recognition of oxoanions: syntheses, titrations, stereoelectronic effects, and selectivity. *J. Am. Chem. Soc.* **2002,** 124, (8), 1782-9.
7. Daniel, M. C.; Astruc, D., Gold nanoparticles: assembly, supramolecular chemistry, quantum-size-related properties, and applications toward biology, catalysis, and nanotechnology. *Chem. Rev.* **2004,** 104, (1), 293-346.
8. Kuo, C. H.; Fruk, L.; Niemeyer, C. M., Addressable DNA-myoglobin photocatalysis. *Chem. Asian J.* **2009,** 4, (7), 1064-9.
9. Onoda, A.; Himiyama, T.; Ohkubo, K.; Fukuzumi, S.; Hayashi, T., Photochemical properties of a myoglobin-CdTe quantum dot conjugate. *Chem. Commun.* **2012,** 48, (65), 8054-6.
10. Weber, G.; Shinitzky, M., Failure of Energy Transfer between Identical Aromatic Molecules on Excitation at the Long Wave Edge of the Absorption Spectrum. *Proc. Natl. Acad. Sci. (U S A)* **1970,** 65, (4), 823-30.
11. Ferguson, B. Q.; Yang, D. C., Methionyl-tRNA synthetase induced 3'-terminal and delocalized conformational transition in tRNAfMet: steady-state fluorescence of tRNA with a single fluorophore. *Biochem.* **1986,** 25, (3), 529-39.
12. Kawski, A., Excitation-Energy Transfer and Its Manifestation in Isotropic Media. *Photochem. Photobiol.* **1983,** 38, (4), 487-508.
13. Gross, E., [27] The cyanogen bromide reaction. In *Methods Enzmol.*, Elsevier: 1967; Vol. 11, pp 238-255.

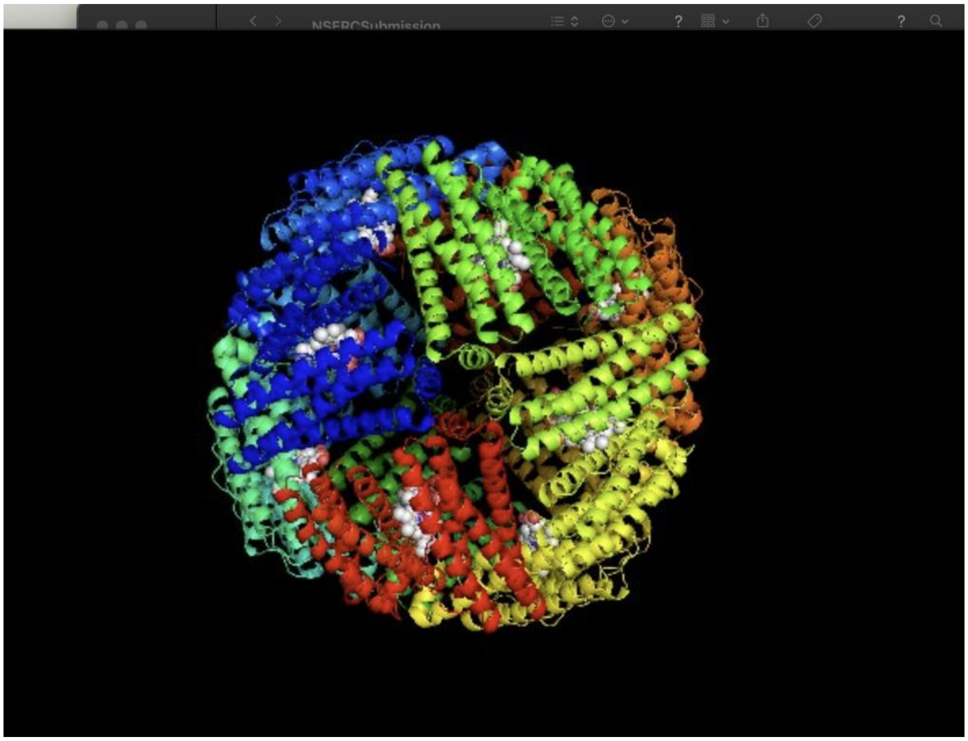

BacterioferritinOverview.mov

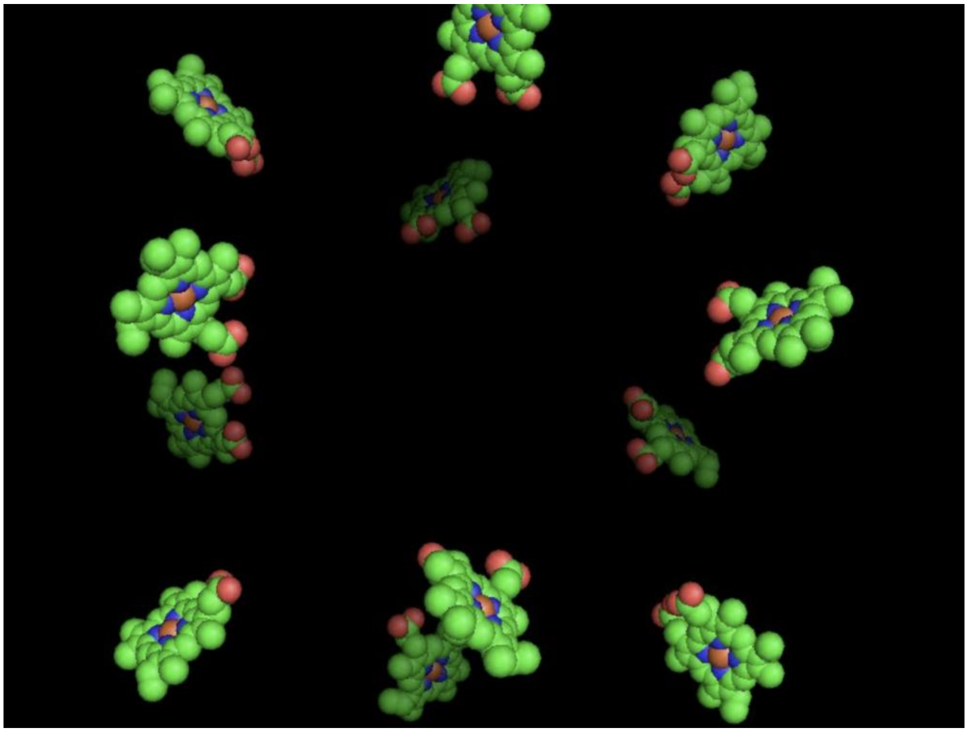

HemeOrientation.mov
